#  

**DOI:** 10.1002/ueg2.12474

**Published:** 2023-11-06

**Breakthroughs in IBD treatment and outcomes: Late‐breaking abstracts**

**Sunday, 15 October 2023 17:00–18:00/A1**

## LB01 | RISANKIZUMAB VERSUS USTEKINUMAB FOR PATIENTS WITH MODERATE TO SEVERE CROHN'S DISEASE: RESULTS FROM THE PHASE 3B SEQUENCE STUDY

L. Peyrin‐Biroulet ^1^, J. C. Chapman^2,3,4^, J.‐F. Colombel^5^, F. Caprioli^6^, G. R. D'Haens^7^, M. Ferrante^8^, S. Schreiber^9^, R. Atreya^10^, S. Danese^11^, J. O. Lindsay^12^, P. Bossuyt^13^, B. Siegmund^14^, P. Irving^15^, R. Panaccione^16^, E. Neimark^17^, K. Wallace^17^, T. Anschutz^17^, K. Kligys^17^, R. Duan^17^, V. Pivorunas^17^, X. Huang^17^, S. Berg^17^, L. Shu^17^, M. C. Dubinsky^18^

^1^Inserm U1256, Nancy University Hospital, Inserm U1256 ‐ Team 2 “Inflammation/Cellular Stress and Exposure to Environmental Risks”, Vandoeuvre‐les‐Nancy, France, ^2^Crohn's and Colitis Center at the Baton Rouge General, Baton Rouge, USA, ^3^GI Alliance, Baton Rouge, USA, ^4^Louisiana State University Health Sciences Center, Baton Rouge, USA, ^5^Gastroenterology, Icahn School of Medicine at Mount Sinai, USA, ^6^Fisiopatologia Medico‐Chirurgica e dei Trapianti, University of Milan, Milano, Italy, ^7^AMC Amsterdam Inflammatory Bowel Disease Centre ‐ Academic Medical Center, AMC Amsterdam Inflammator, Academic Medical Center, Amsterdam, Netherlands, ^8^Department of Gastroenterology and Hepatology, University Hospitals Leuven, Leuven, Belgium, ^9^Department of Internal Medicine I, University Hospital Schleswig‐Holstein, Kiel, Germany, ^10^Medical Department 1, University Erlangen‐Nuremberg, Erlangen, Germany, ^11^Gastrointestinal Immunopathology, Vita‐Salute San Raffaele University ‐ IRCCS San Raffaele Scientific Institute, Milan, Netherlands, ^12^Digestive Disorders Clinical Academic Unit, Department of Digestive Diseases, Barts & The London School of Medicine, London, UK, ^13^Imelda General Hospital, Bonheiden, Belgium, ^14^Charité ‐ Universitätsmedizin Berlin, Med. Klinik m.S. Gastroenterologie, Infektiologie und Rheumatologie, Berlin, Germany, ^15^Department of Gastroenterology, Guy's and St Thomas' Hospital, London, UK, ^16^Medicine, University of Calgary, Calgary, Canada, ^17^AbbVie, North Chicago, USA, ^18^Mount Sinai Hospital, Mount Sinai, New York City, USA

**Contact E‐Mail Address**: peyrinbiroulet@gmail.com

**Introduction**: Inhibition of interleukin (IL)‐23 has been demonstrated as a safe and effective mechanism of action for treating various chronic inflammatory diseases, including Crohn's disease (CD). The phase 3b SEQUENCE study directly compared the efficacy and safety of risankizumab (RZB), a selective IL‐23 p19 subunit inhibitor, and ustekinumab (UST), an IL‐12/IL‐23 p40 subunit inhibitor, in patients (pts) with moderate to severe CD.

**Aims & Methods**: SEQUENCE, an open‐label, multicenter, randomized, efficacy assessment‐blinded study, enrolled pts who previously failed ≥1 anti‐TNF therapies and had a CD Activity Index score of 220–450, Simple Endoscopic Score for CD ≥6 for ileocolonic or colonic disease (≥4 for isolated ileal disease) excluding the presence of narrowing component, plus an average (avg) daily stool frequency ≥4 and/or avg daily abdominal pain score ≥2. In Part 1 of the trial, described herein, pts were randomized 1:1 to receive RZB (600 mg IV induction dosing at baseline [BL], weeks 4 and 8, followed by 360 mg SC maintenance dosing every 8 weeks [Q8w] starting at week 12) or UST (a single weight‐based IV induction dose, followed by 90 mg SC maintenance dosing Q8w starting at week 8) over a period of 48 weeks.^1^ Randomization was stratified by number of anti‐TNF therapies failed (1, >1) and steroid use at BL (Y, N). A mandatory steroid taper started at week 2. Two primary endpoints were clinical remission at week 24 (non‐inferiority of RZB vs. UST in 50% of planned subjects) and endoscopic remission at week 48 (superiority of RZB vs. UST). Ranked secondary endpoints included clinical remission at week 48, endoscopic response at weeks 48 and 24, steroid‐free (SF) endoscopic remission at week 48, and SF clinical remission at week 48 (all tested for superiority of RZB vs. UST). Safety was assessed throughout the trial.

**Results**:

**Table:** 

Variable	RZB	UST	Treatment difference
	*N* = 255	*N* = 265	RZB—UST
	*n*/*N* (%)	*n*/*N* (%)	% [95% CI]
Primary^a^			
1. Clinical remission at wk 24 (ITT‐1H^b^)	75/128 (58.6%)	54/137 (39.5%)	Non‐inferiority^c^ met; 18.4 [6.6, 30.3]
2. Endoscopic remission at wk 48 (ITT‐1^d^)	81/255 (31.8%)	43/265 (16.2%)	15.6 [8.4, 22.9], *p* < 0.0001
Ranked secondary^a,d^			
1. Clinical remission at wk 48	155/255 (60.8%)	108/265 (40.8%)	19.7 [11.3, 28.1], *p* < 0.0001
2. Endoscopic response at wk 48	115/255 (45.1%)	58/265 (21.9%)	23.3 [15.4, 31.2], *p* < 0.0001
3. Endoscopic response at wk 24	115/255 (45.2%)	70/265 (26.4%)	18.9 [10.9, 26.9], *p* < 0.0001
4. Steroid‐free endoscopic remission at wk 48	80/255 (31.4%)	41/265 (15.5%)	15.9 [8.8, 23.1], *p* < 0.0001
5. Steroid‐free clinical remission at wk 48	155/255 (60.8%)	107/265 (40.4%)	20.1 [11.7, 28.4], *p* < 0.0001

Treatment emergent adverse events (AEs)^e^	RZB	UST	Treatment difference
	*N* = 262	*N* = 265	RZB—UST
	PYs = 257.6	PYs = 269.9	% [95% CI]
	Events (E/100 PYs)^f^	Events (E/100 PYs)^f^	
All TEAEs	879 (341.2)	763 (282.7)	58.5 (28.3, 88.7)
Investigator‐defined drug‐related AE^g,h^	167 (64.8)	111 (41.1)	23.7 (11.2, 36.2)
Severe AE	60 (23.3)	82 (30.4)	−7.1 (−15.9, 1.7)
Serious AE	36 (14.0)	64 (23.7)	−9.7 (−17.1, −2.4)
AEs leading to study drug discontinuation	10 (3.9)	14 (5.2)	−1.3 (−4.9, 2.3)
Death	0	0	0
Adverse events of special interest			
Adjudicated MACE/extended MACE^i^	0	1 (0.4)	−0.4 (−1.1, 0.4)
Serious infections	10 (3.9)	14 (5.2)	−1.3 (−4.9, 2.3)
Active tuberculosis	0	0	0
Opportunistic infections excluding tuberculosis and herpes zoster^j^	1 (0.4)	0	0.4 (−0.4, 1.1)
Herpes zoster	1 (0.4)	1 (0.4)	0.0 (−1.0, 1.1)
Malignant tumours^k^	1 (0.4)	1 (0.4)	0.0 (−1.0, 1.1)
Non‐melanoma skin cancer (NMSC)	1 (0.4)	0	0.4 (−0.4, 1.1)
Malignancies excluding NMSC	0	1 (0.4)	−0.4 (−1.1, 0.4)
Hypersensitivity	37 (14.4)	32 (11.9)	2.5 (−3.7, 8.7)
Serious hypersensitivity	0	0	0
Adjudicated anaphylactic reaction	0	0	0
Hepatic events	26 (10.1)	23 (8.5)i	1.6 (−3.6, 6.8)
Injection site reactions	5 (1.9)	8 (3.0)	−1.0 (−3.7, 1.6)

Abbreviations: AE, adverse event; BL, baseline; CD, Crohn's disease; CDAI, CD activity index; ITT, intention to treat; RZB, risankizumab; PY, patient years; SES‐CD, simple endoscopic score for CD; TEAE, treatment emergent AE; UST, ustekinumab; wk, week.

Clinical remission – CDAI <150.

Endoscopic remission ‐ SES‐CD ≤ 4 and at least a 2‐point reduction versus BL and no sub score >1 in any individual variable, as scored by a central reviewer blinded to treatment allocation.

Endoscopic response ‐ decrease in SES‐CD > 50% from BL (or for pts with isolated ileal disease and a BL SES‐CD of 4, at least a 2‐point reduction from BL), as scored by central reviewer.

Steroid‐free endoscopic remission ‐ endoscopic remission and not receiving steroids at the corresponding visit.

Steroid‐free clinical remission ‐ clinical remission and not receiving steroids at the corresponding visit.

*SEQUENCE Part 1 compared the efficacy and safety of RZB versus UST over 48 weeks, while Part 2 is an ongoing open‐label long‐term extension to evaluate the long‐term safety of RZB in pts who received RZB in Part 1 and completed week 48 visit.

^a^Categorical variables were analyzed using Cochran‐Mantel‐Haenszel (CMH) test. For both the primary and secondary endpoints, the non‐responder imputation while incorporating multiple imputation to handle missing data (due to COVID‐19 or geopolitical conflict) was used. Primary and secondary endpoints were tested sequentially in the order specified using the CMH risk difference estimate test stratified by the number of failed anti‐TNF therapies (1, >1) and steroid use at baseline (yes, no).

^b^ITT1H population is a subset of ITT‐1 population and includes approximately 50% of ITT1 subjects who have opportunity to reach week 24 by the time of the primary analysis of CDAI clinical remission at week 24; non‐inferiority of RZB versus UST.

^c^Non‐inferiority test: If the lower limit of 95% confidence interval based on the CMH estimation for the risk difference between RZB and UST groups (RZB – UST) was greater than the negative of 10% noninferiority margin, then noninferiority was demonstrated for clinical remission at week 24. If non‐inferiority was demonstrated for clinical remission (CDAI) at Week 24, the superiority for the endoscopic remission at Week 48 was subsequentially tested at two‐sided significance level of 0.05 using the CMH test.

^d^Intent‐to‐treat‐1 (ITT‐1) population included all randomized subjects who were randomized to RZB with the selected RZB dose (RZB 600 mg IV followed by RZB 360 mg SC) or UST and who received at least 1 dose of study drug during Part 1 of the study; superiority of RZB versus UST.

^e^TEAEs are events that begin either on or after the first dose of UST or RZB in Part 1 and until the first dose of study drug (RZB) in Part 2 if the pt is enrolled into the Part 2 or within 140 days after the last dose administration of the study drugs (UST or RZB) in Part 1 if the pt does not participate in Part 2. Only safety data obtained before 12JUL2023 or Week 52 dosing date (the first dose date of Part 2), whichever occurred earlier, are reported here.

^f^SA1 population includes all pts who received at least 1 dose of study drug during Part 1 of the study.

^g^As assessed by investigator.

^h^RZB related: 3 patients with SAEs related to RZB (anal fistula, anal abscess, campylobacter, cystitis, localized infection, genital fistula); 8 patients with SAEs related to UST (abdominal pain, anal fistula, CD, ileal stenosis, vomiting).

^i^MACE: Major Adverse Cardiovascular Events, defined as cardiovascular death or death due to stroke, non‐fatal myocardial infarction, and non‐fatal stroke; extended MACE defined as MACE with hospitalization for unstable angina and coronary revascularization procedures.

^j^Opportunistic infection: RZB, oesophageal candidiasis.

^k^Malignant tumor: RZB, squamous cell carcinoma of skin; UST, anal squamous cell carcinoma.

^l^One case of potential Hy's Law in the UST treatment group.

In total, 520 pts were randomized and assessed for efficacy: RZB (*N* = 255) and UST (*N* = 265). The proportion of pts who completed the study was numerically higher with RZB (89.4%) versus UST (74.0%). The two primary endpoints of the study were met. At week 24, in half of the patients enrolled, clinical remission rates were 58.6% (75/128) for RZB and 39.5% (54/137) for UST (Δ18.4 [6.6–30.3], non‐inferiority met with the pre‐defined margin of 10%) (Table). At week 48, endoscopic remission rates were 31.8% (81/255) for RZB and 16.2% (43/265) for UST (Δ15.6% [8.4–22.9], *p* < 0.0001 for superiority). RZB was superior to UST for all secondary endpoints (all *p* < 0.0001). Exposure adjusted adverse event (AE) rates (Events [E/100 PYs]) were comparable between RZB (879 [341.2]) and UST (763 [282.7]). Rates of serious AEs and AEs leading to study drug discontinuation were numerically higher with UST than RZB (Table). Rates of serious infections and hepatic events were similar between treatment groups, with no serious hepatic events with RZB. Two cases of malignancy were reported (one in each treatment arm). There was one event of adjudicated MACE with UST. There were no deaths.

**Conclusion**: In pts with moderate to severe CD who failed anti‐TNF therapy, RZB demonstrated non‐inferiority to UST in achieving week 24 clinical remission, superiority in achieving week 48 endoscopic remission, and superiority for achieving all secondary endpoints. The safety profiles of RZB and UST were consistent with previously published results.

**Reference**

1. Stelara Label [Internet]. Available from: https://www.accessdata.fda.gov/drugsatfda_docs/label/2022/125261s161lbl.pdf

**Disclosure**: Laurent Peyrin‐Biroulet: Fees: Galapagos, AbbVie, Janssen, Genentech, Alimentiv, Ferring, Tillots, Celltrion, Takeda, Pfizer, Index, Sandoz, Celgene, Biogen, SamsungBioepis, Inotrem, Allergan, MSD, Roche, Arena, Gilead, Amgen, BMS, Vifor, Norgine, Mylan, Lilly, Fresenius Kabi, OSEImmunotherapeutics, Enthera, Theravance, Pandion, Gossamer, Viatris, ThermoFisher, ONOPharma, Mopac, Cytoki, Morphic, Prometheus, Applied MolecularTransport; J. Casey Chapman: Consultant: AbbVie, Medtronic; Advisory Board: AbbVie, Pfizer, Takeda; Speaker: AbbVie, BMS, Janssen, Pfizer, Takeda; Jean‐Frederic Colombel: Grants: AbbVie, Janssen, Takeda, BMS; Speaking fees: AbbVie, Takeda; Consultant: AbbVie, Amgen, AnaptysBio, Allergan, Arena, BI, BMS, Celgene, Celltrion, Lilly, Ferring, Galmed, GSK, Genentech (Roche), Janssen, Kaleido, Immunic, IterativeScopes, Merck, Landos, Microba Life Science, Novartis, Otsuka, Pfizer, ProtagonistTherapeutics, Sanofi, Takeda, TiGenix, Vifor; Stock options: IntestinalBiotechDevelopment; Flavio Caprioli: Consultant: AbbVie, MSD, Takeda, Janssen, Roche, Celgene, BMS, Galapagos, Gllead, Pfizer, Mundipharma, Galapagos, Biogen; Speaker: AbbVie, Ferring, Takeda, AllergyTherapeutics, Janssen, Pfizer, Biogen; Research grants: Giuliani, Sofar, MSD, Takeda, AbbVie; Geert D’Haens: Advisor: AbbVie, Ablynx, ActiveBiotechAB, AgomabTherapeutics, Alimentiv, Allergan, Alphabiomics, Amakem, Amgen, AMPharma, AppliedMolecularTherapeutics, Arena, AstraZeneca, Biogen, BMS, BI, Celltrion, DSMPharma; Echo, Engene, Exeliom, Ferring, Dr Falk, Galapagos, Genentech/Roche, Gilead, GSK, Gossamerbio, Immunic, J&J, Kintai, Lilly, Lument, Lycera, Medtronic, MSD, MitsubishiTanabe, NovoNordisk, Otsuka, Pfizer, Photopill, ProciseDx, Prodigest, Progenity, Prometheus/Nestlé, Protagonist, RedHill, SamsungBioepis, Sandoz, Seres, Setpoint, Shire, Takeda, Teva, Tillotts, Topivert, Versant, Vifor. Marc Ferrante: Grants: AbbVie, Amgen, Biogen, EG, Janssen, Pfizer, Takeda, Viatris; Consultant: AbbVie, Agomab, BI, Celgene, Celltrion, Lilly, Janssen‐Cilag, Medtronic, MSD, Pfizer, Regeneron, SamsungBioepis, Sandoz, Takeda, ThermoFisher; Speaker: AbbVie, Amgen, Biogen, BI, Falk, Ferring, Janssen‐Cilag, Lamepro, MSD, Pfizer, Sandoz, Takeda, Truvion, Viatris; Stefan Schreiber: Consultant: AbbVie, Amgen, Arena, BMS, BI, Celltrion, Dr Falk, Ferring, Fresenius, Galapagos, Genentech, Gilead, GSK, IMAB‐Biopharma, Janssen, Lilly, Merck, Novartis/Sandoz, Pfizer, Protagonist, Takeda, Theravance. Raja Atreya: Speaker, consultant, or grants: AbbVie, Amgen, Arena, Biogen, BI, BMS, Cellgene, Celltrion, DrFalk, Galapagos, Gilead, InDex, Janssen‐Cilag, Lilly, MSD, Novartis, Pandion, Pfizer, Roche, SamsungBioepis, Takeda, Viatris; Silvio Danese: Consultant: AbbVie, Alimentiv, Allergan, Amgen, AstraZeneca, AthosTherapeutics, Biogen, BI, Celgene, Celltrion, Lilly, Enthera, Ferring, Gilead, Hospira, Inotrem, Janssen, J&J, MSD, Mundipharma, Mylan, Pfizer, Roche, Sandoz, Sublimity, Takeda, TiGenix, UCB, and Vifor; Lecture fees: Abbvie, Amgen, Dr Falk, Ferring, Gilead, Janssen, Mylan, Pfizer, Takeda. James O. Lindsay: Consultant/advisory board: AbbVie, AtlanticHealthcare, BMS, Celgene, Celltrion, Lilly, Ferring, Galapagos, Gilead, GSK, Janssen, MSD, Napp, Pfizer, Shire, Takeda, Vifor; Speaker fees/sponsorship: AbbVie, Ferring, Janssen, MSD, Napp, Norgine, Pfizer, Shire, Tillotts, Takeda; Grants: AbbVie, Gilead, Pfizer, Shire, Takeda; Peter Bossuyt: Grants: AbbVie, Amgen, Janssen, Mundipharma, Mylan, Pfizer; Speaker: AbbVie, Celltrion, Janssen, Pfizer, Takeda; Advisory Board: AbbVie, Arena, BMS, Hospira, Janssen, Lilly, Merck, Mundipharma, PentaxMedical, Pfizer, PSI CRO, Roche, Sandoz, Takeda; Britta Siegmund: Consultant: AbbVie, Arena, BI, BMS, Celgene, Falk, Galapagos, Janssen, Lilly, Pfizer, Prometheus, Takeda; Speaker's fees: AbbVie, CEDServiceGmbH, Falk, Ferring, Janssen, Novartis, Pfizer, Takeda; Peter Irving: Grants/Fees: Janssen, MSD,Takeda; Personal fees: AbbVie, Arena, Dr. Falk, Lilly, Ferring, Genentech, Hospira, Pfizer, Pharmacosmos, SamsungBioepis, Sandoz, Shire, Tillotts, TopiVert, VHsquared, Vifor, WarnerChilcott. Remo Panaccione: Consultant: AbbVie, Abbott, Alimentiv, Amgen, Arena, AstraZeneca, BMS, BI, Celgene, Celltrion, Cosmos, Eisai, Elan, Ferring, Galapagos, Genentech, Gilead, GSK, Janssen, Lilly, Merck, Mylan, Oppilan, Pandion, Pfizer, Progenity, Protagonist, Roche, Sandoz, Satisfai, Schering‐Plough, Shire, Sublimity, Theravance, UCB, Takeda; Speaker: AbbVie, Arena, Celgene, Ferring, Gilead, Janssen, Lilly, Merck, Pfizer, Roche, Sandoz, Shire, and Takeda; Grants: AbbVie, Ferring, Janssen, Pfizer, Takeda; Advisory board: AbbVie, Amgen, Arena, BMS, Celgene, Celltrion, Ferring, Galapagos, Genentech, Gilead, GSK, Janssen, Lilly, Merck, Mylan, Oppilan, Pandion, Pfizer, Sandoz, Shire, Sublimity, Takeda, Theravance. Marla Dubinsky: Consultant: AbbVie, Arena, BMS, Celgene, Lilly, Genentech, Gilead, Janssen, Pfizer, Prometheus, Takeda; Founder/shareholder Trellus Health. Ezequiel Neimark, Kori Wallace, Toni Anschutz, Kristina Kligys, W Rachel Duan, Valerie Pivorunas, Xiu Huang, Sofie Berg, Lei Shu: AbbVie full‐time employees; may own AbbVie stock or options.

## LB02 | A RETROSPECTIVE COHORT STUDY TO COMPARE THE EFFICACY, PHARMACOKINETICS AND SAFETY OF SUBCUTANEOUS (SC) AND INTRAVENOUS (IV) INFLIXIMAB IN THE TREATMENT OF INFLAMMATORY BOWEL DISEASE

T. Mahmood ^1^, J. O. Lindsay^2^, A. Ibarra^2^

^1^Medicine, Barts and the London, Queen Mary University of London, London, UK, ^2^Digestive Disorders Clinical Academic Unit, Department of Digestive Diseases, Barts & the London School of Medicine, London, UK

**Contact E‐Mail Address**: tahamahmood6480@gmail.com

**Introduction**: Only a small number of trials have been conducted on the use of SC Infliximab in IBD with a lack of data to directly compare with IV Infliximab. These trials have focused upon patients newly started on Infliximab. There has been an under representation of patients who have been established on IV Infliximab for a longer period of time and then undergone SC switch as well as higher risk patient groups including those with Perianal Disease and Escalated Dosing Regimens of IV Infliximab. We conducted the largest study completed so far in the UK comparing the efficacy, pharmacokinetics and safety of SC with IV Infliximab in IBD. The novelty of SC Infliximab use in the UK combined with the scarcity of units currently using this treatment and critical study outcomes at 1 year only becoming available in May 2023 have prompted us to submit this as a late breaking abstract.

**Aims & Methods**: Retrospective cohort study of 306 patients in established clinical remission receiving Infliximab at an IBD centre in the UK during May 2023. Efficacy was assessed using objective biomarkers (CRP, FCP), clinical disease activity scores (HBI/SCCAI) at Baseline, 3–6, 12–15 months and treatment persistence. Pharmacokinetics was assessed through treatment discontinuation secondary to Anti‐Drug Antibody formation and changes in serum drug levels. Safety was assessed through the frequency of treatment related side effects. Statistical analyses included; Descriptive, Wilcoxon, ANOVA, Regression, KMC/Log Rank and MWU Test. Ethical approval was not required.

**Results**: 199 patients were on SC Infliximab and 107 patients on IV Infliximab. The Efficacy of SC Infliximab was demonstrated through no significant change in the CRP and FCP from baseline when compared to values at 3–6 months and 12–15 months (CRP [*p* = 0.344]) (FCP [*p* = 0.301]). Higher rates of treatment persistence were reported on SC Infliximab when compared to the IV group (87% SC vs. 35% IV, *p* < 0.001). This difference persisted when escalation of the IV dose was excluded as the reason for the failure of treatment persistence (87% SC vs. 76% IV, *p* = 0.001). An increase in drug levels on treatment was found to be a significant factor for treatment persistence in the SC group (*p* = 0.001) (OR 31.77). However, perianal disease and the dosing of IV Infliximab prior to SC switch were not significant factors for treatment persistence in the SC group (Perianal Disease [*p* = 0.757] [OR 1.2]) (Dosing of IV Infliximab [*p* = 0.513] [OR 0.67]). There were significant differences between the SC and IV Infliximab group when comparing drug levels on treatment (*p* < 0.001), the CRP at 3–6/12 (*p* < 0.001) and the FCP at 3–6/12 (*p* < 0.001). Those on SC Infliximab had a significant increase in drug levels on treatment not observed in the IV group with higher drug levels and more frequent increase in drug levels (SC [*p* < 0.001] [Median 6 Before, Median 14 After] [83.2%]), (IV [*p* = 0.085] [Median 3.1 1st, Median 4.8 2nd] [54.4%]). Furthermore, Anti‐Drug Antibody (ADA) formation leading to treatment discontinuation was less frequently observed on SC Infliximab (2% SC vs. 20% IV). Treatment discontinuation due to side effects was less frequent in those on SC Infliximab (3% SC vs. 13% IV).

**Conclusion**: SC switch should be considered in patients who are in established clinical remission on IV Infliximab as offering an effective and safe alternative, even in higher risk patients, with an increase in serum drug levels predictive of treatment success. The SC route displays different pharmacokinetic properties to IV which are favourable given the lower rates of treatment discontinuation.

**References**

1. Peyrin‐Biroulet L, Arkkila P, Armuzzi A, Danese S, Guardiola J, Jahnsen J, et al. Comparative efficacy and safety of infliximab and vedolizumab therapy in patients with inflammatory bowel disease: a systematic review and meta‐analysis. *BMC Gastroenterol*. 2022;22(1). 10.1186/s12876‐022‐02347‐1
2. Bots SJ, Parker CE, Brandse JF, Löwenberg M, Feagan BG, Sandborn WJ, et al. Anti‐drug antibody formation against biologic agents in inflammatory bowel disease: a systematic review and meta‐analysis. *BioDrugs* 2021;35(6):715–733. 10.1007/s40259‐021‐00507‐5
3. Haar GS, Vasudevan A, Curtain CM, van Langenberg DR. Assessing adherence to infusion‐based biologic therapies in patients with inflammatory bowel disease. *Res Soc Adm Pharm*. 2021;17(8):1420–1425. 10.1016/j.sapharm.2020.10.011
4. Schreiber S, Ben‐Horin S, Leszczyszyn J, Dudkowiak R, Lahat A, Gawdis‐Wojnarska B, et al. Randomized controlled trial: subcutaneous vs intravenous infliximab CT‐P13 maintenance in inflammatory bowel disease. *Gastroenterology* 2021;160(7):2340–2353. 10.1053/j.gastro.2021.02.068
5. Buisson A, Nachury M, Reymond M, Yzet C, Wils P, Payen L, et al. Effectiveness of switching from intravenous to subcutaneous infliximab in patients with inflammatory bowel diseases: the REMSWITCH study. *Clin Gastroenterol Hepatol*. 2022. [Preprint]. 10.1016/j.cgh.2022.08.011
6. Smith PJ, Critchley L, Storey D, Gregg B, Stenson J, Kneebone A, et al. Efficacy and safety of elective switching from intravenous to subcutaneous infliximab [CT‐P13]: a multicentre cohort study. *J Crohns Colitis* 2022;16(9):1436–1446. 10.1093/ecco‐jcc/jjac053

**Disclosure**: Nothing to disclose

## LB03 | THE LONG‐ACTING GLP‐2 ANALOG APRAGLUTIDE PROVIDES CLINICAL BENEFIT FOR PATIENTS WITH SHORT BOWEL SYNDROME WITH INTESTINAL FAILURE AND COLON IN CONTINUITY AT 52 WEEKS

A. Verbiest ^1,2^, M. K. Hvistendahl^3^, F. Bolognani^4^, C. Li^4^, N. N. Youssef^5^, P. B. Jeppesen^3^, F. Joly^6^, T. Vanuytsel^1,2^

^1^Department of Chronic Diseases and Metabolism (ChroMetA), Translational Research Center for Gastrointestinal Disorders (TARGID), University of Leuven, Leuven, Belgium, ^2^Leuven Intestinal Failure and Transplantation Center (LIFT), University Hospitals Leuven, Leuven, Belgium, ^3^Department of Intestinal Failure and Liver Diseases, Rigshospitalet, Copenhagen University Hospital, Copenhagen, Denmark, ^4^Clinical Development, VectivBio AG, Basel, Switzerland, ^5^Medical Affairs, VectivBio AG, Basel, Switzerland, ^6^Department of Gastroenterology and Nutritional Support, Centre for Intestinal Failure, Hôpital Beaujon, Clichy, France

**Contact E‐Mail Address**: astrid.verbiest@kuleuven.be

**Introduction**: Short bowel syndrome (SBS) is a rare gastrointestinal condition with a high risk of developing intestinal failure (SBS‐IF) and need for parenteral support (PS). Glucagon‐like peptide‐2 (GLP‐2) analogs stimulate adaptation of the remaining gut resulting in increased intestinal absorption and reduced PS needs. Extensive literature is available on the effect of teduglutide in patients (pts) without a remaining colon. However, in SBS‐IF with a colon‐in‐continuity (CiC), where there is less dependency on IV fluid compared to calories, the impact of GLP‐2 analogs is much less established. Apraglutide (APRA) is a novel, long‐acting GLP‐2 analog in development for SBS‐IF.

**Aims & Methods**: This multicenter, open‐label, phase 2 study in SBS‐IF‐CiC aims to investigate the safety and efficacy of APRA in reducing PS needs at 52 weeks. STARS Nutrition is a 52‐w study in adult pts with SBS‐IF‐CiC receiving once‐weekly subcutaneous APRA injections (5 mg). Metabolic balance studies were performed at baseline and 4 weeks with stable PS, followed by a 48‐week PS adjustment period. During monthly 48 h fluid balance assessments, oral fluid intake was kept constant by adhering to an individual predefined drinking menu while urine was collected. PS was reduced according to a pre‐defined algorithm if an increase in mean daily urinary output of ≥10% was observed. Safety was the primary endpoint. Secondary endpoints included changes in weekly PS volume, energy content, and PS days, and proportion of clinical responders (PS reduction of ≥20%). Data are presented as mean (95% CI) unless specified otherwise. Nominal *p*‐values are calculated using paired *T*‐tests with significance set at 0.05.

**Results**: Nine pts were included and comprise the full study population. Small bowel length was 19 (range 0–50) cm and 79 (range 43–100) % of the colon was in continuity. Pts had baseline PS needs of 10 (range 4–21) L distributed over 5.2 (range 3–7) days/week. APRA had an acceptable safety profile, consistent with previous data. All pts experienced ≥1 adverse event (AE) after initiating APRA; a total of 127 AEs was reported. Three pts experienced serious AEs. APRA was interrupted for 1 week in one patient with acute cholangitis; APRA was restarted and successfully continued to study completion. PS‐related outcomes are presented in Table 1. Absolute weekly PS volume decreased significantly by −4702 (−6539 to −2865) mL/week (*p* = 0.0004). Accordingly, PS energy content significantly decreased by −3510 (−4991 to −2029) kcal/week (*p* = 0.0006). All pts qualified as clinical responders. Seven pts (78%) achieved ≥1 day off PS of whom two pts (22%) reached enteral autonomy. Pts gained an additional 2.1 (0.7–3.6) days off per week. Body weight and daily urine output remained constant despite PS reductions.

**TABLE 1:** Parenteral support related outcomes after 24 and 52 weeks of apraglutide treatment; mean (95% CI); *p < 0.05.

	Baseline	24 weeks	52 weeks
Actual weekly PS volume (mL/week)	9919 (5791–14,047)	6409 (2721–10,096)	5217 (1935–8499)
		*p* = 0.0005*	*p* = 0.0004*
PS energy content (kcal/week)	7938 (4832–11,044)	5174 (2505–7844)	4428 (2236–6620)
		*p* = 0.0019*	*p* = 0.0006*
Weight (kg)	57.7 (54.1–61.3)	59.3 (55.5–63.1)	55.9 (51.7–60.2)
		*p* = 0.0327*	*p* = 0.1355
Oral fluid intake (mL/day)	1604 (1335–1874)	1604 (1335–1874)	1604 (1335–1874)
Daily urinary output (mL)	1511 (1182–1840)	1673 (1178–2168)	1453 (1121–1785)
		*p* = 0.2397	*p* = 0.5956

**Conclusion**: APRA has an acceptable safety profile and significantly reduces PS needs in pts with SBS‐IF‐CiC resulting in days without PS. STARS Nutrition is the first study prospectively showing evidence for clinical benefit of a long‐acting GLP‐2 analog in SBS‐IF‐CiC.

**Disclosure**: PBJ has received grants/research support/honoraria or consultation fees from Albumedix A/S, ArTara Therapeutics, Bainan Biotech, Baxter, Coloplast, Ferring, Fresenius Kabi, GLyPharma, Naia Pharma, NPS Pharmaceuticals, Shire, Takeda, The Novo Nordisk Foundation, Therachon, VectivBio and Zealand Pharma.

FJ has received grants/research support/honoraria or consultation fees from Baxter, Fresenius Kabi, Nestlé Health Sciences, BBraun, Theradial, Mayoli, Biocodex, mobile3e Consulting, Carembouche, NPS Pharmaceuticals, Shire, Takeda, Therachon, VectivBio and Zealand Pharma.

TV has served on the speaker bureau for Fresenius Kabi, Remedus, Takeda, VectivBio and Zealand Pharma. TV has provided clinical advice to Baxter, Shire, Takeda, VectivBio, Zealand Pharma. TV has received research grants from Takeda and VectivBio.

This research was supported by VectivBio. FB, CL and NNY are employees of VectivBio.

## LB04 | INFLAMMATORY BOWEL DISEASE: A PHENOME‐WIDE PRE‐ AND POST‐DIAGNOSTIC ASSOCIATION STUDY

A. Ebert^1^, R. Elmahdi ^1,2^, G. Poulsen^1^, M. Bøgsted^1^, B. Verstockt^3^, C. W. Lees^4^, T. Jess^1,2^

^1^Center for Molecular Prediction of Inflammatory Bowel Disease, Aalborg University, PREDICT; Department of Clinical Medicine, København, Denmark, ^2^Department of Gastroenterology & Hepatology, Aalborg University Hospital, Aalborg, Denmark, ^3^Department of Gastroenterology and Hepatology and Department of Chronic Diseases, Metabolism and Aging, Translational Research in Gastrointestinal Disorders ‐ IB, University Hospitals Leuven and KU Leuven, Leuven, Belgium, ^4^Edinburgh IBD Unit, University of Edinburgh, Edinburgh, UK

**Contact E‐Mail Address**: re107@ic.ac.uk

**Introduction**: Inflammatory bowel disease (IBD), including Crohn's disease (CD) and ulcerative colitis (UC), is known to be associated with various extra‐intestinal manifestations, impacting organ systems beyond the gastrointestinal tract. Identifying comorbidities in IBD and the timing of their development can provide valuable insight into the mechanisms underlying IBD development.

**Aims & Methods**: We conducted the first population‐ and disease‐wide phenomic association study in IBD, using >6 million ICD‐10 coded healthcare contacts from 10 years before and up to 17 years after IBD diagnosis to investigate associations with 1583 diseases. In order to explore co‐morbidities with potential aetiological significance with IBD, we additionally assessed the strength of association with all diseases in the pre‐diagnosis compared with the post‐diagnosis period. To correct for multiple testing, we adjust our significance threshold (*p* < 0.05) with the Bonferroni correction, resulting in an adjusted *p*‐value of 7.90 × 10^−6^, which we refer to as disease‐wide statistical significance.

**Results**: We identified 312 disease associations with disease‐wide statisitical significance and 125 of these diseases appear up to 10 years before diagnosis. The risk of immune‐mediated diseases and extra‐intestinal manifestations are among those diseases increased up to 10 years prior to IBD diagnosis (psoriasis: RR_CD_: 2.57, 95% CI: 2.00–3.29; RR_UC_: 1.54, 95% CI: 1.25–1.87; enteropathic arthropathies: RR_CD_: 3.57, 95% CI: 2.65–4.78; RR_UC_: 1.8, 95% CI: 1.38–2.32). This was also the case for gastroenterological and liver disorders (gall stones: RR_CD_: 1.82, 95% CI: 1.62–2.04; RR_UC_: 1.26, 95% CI: 1.15–1.37; acute pancreatitis: RR_CD_: 1.83, 95% CI: 1.30–2.53; RR_UC_: 2.27, 95% CI: 1.84–2.79). The risk of cardiometabolic diseases and neuropsychological disorders had increased disease‐wide statistical significance both pre‐ and post‐diagnostically, whereas potential sequelae of treatment, such as osteoporosis (HR_CD_: 2.56, 95% CI: 2.30–2.86; HR_UC_: 1.92, 95% CI: 1.79–2.07) or herpes simplex infections (HR_CD_: 4.04, 95% CI: 2.76–5.91; HR_UC_: 1.69, 95% CI: 1.2–2.38) were primarily seen post‐diagnostically. Of potential aetiological importance to CD, diagnosis with infectious mononucleosis (RR: 1.87, 95% CI: 1.37–2.52) was limited to the pre‐diagnostic period.

**Conclusion**: Our results demonstrate IBD as an essentially multisystemic disease, particularly manifesting as gastrointestinal, metabolic, immune, and neuropsychological disorders, present up to 10 years prior to IBD diagnosis.

**Disclosure**: **AE, GP, and MB** report no competing interests.

**BV** reports research support from AbbVie, Biora Therapeutics, Landos, Pfizer, Sossei Heptares and Takeda; speaker fees from Abbvie, Biogen, Bristol Myers Squibb, Celltrion, Chiesi, Falk, Ferring, Galapagos, Janssen, MSD, Pfizer, R‐Biopharm, Takeda, Truvion and Viatris; consultancy fees from Abbvie, Alimentiv, Applied Strategic, Atheneum, Biora Therapeutics, Bristol Myers Squibb, Galapagos, Guidepont, Landos, Mylan, Inotrem, Ipsos, Janssen, Pfizer, Progenity, Sandoz, Sosei Heptares, Takeda, Tillots Pharma and Viatris.

**RE** reports support from Novo Nordisk.

**CWL** has acted as a consultant to Abbvie, Janssen, Takeda, Pfizer, Galapagos, Bristol 468 Myers Squibb, B.I., Sandoz, Novartis, GSK, Gilead, ViforPharma, Dr Falk, Trellus Health 469 and Iterative Scopes; he has received speaking fees and travel support from Pfizer, 470 Janssen, Abbvie, Galapagos, MSD, Takeda, Shire, Ferring, Hospira and Dr Falk.

## LB05 | THE DISEASE COURSE OF MICROSCOPIC COLITIS – A 5‐YEAR PROSPECTIVE EUROPEAN INCIDENCE COHORT (PRO‐MC)

B. Verhaegh ^1^, A. Münch^2^, S. Wildt^3^, W. Cebula^4^, A.‐R. Diac^5^, M. Al‐khalaf^6^, N. Pedersen^7^, D. Guagnozzi^8^, J. Kupcinskas^9^, V. Kiudelis^10^, F. Fernández‐Bañares^11^, G. E. Tontini^12^, I. Lyutakov^13^, G. Macaigne^14^, S. Miehlke^15^, H. Hjortswang^16^, L. K. Munck^17^

^1^Gastroenterology‐Hepatology, Maastricht University Medical Center, Maastricht, Netherlands, ^2^Department of Gastroenterology and ‐ Gastroenterology, Department of Gastroenterology, Linköping, Sweden, ^3^Department of Gastroenterology, Koege University Hospital, Køge, Denmark, ^4^@Region Sjaelland Nykoebing F. Hospital, Nykoebing F., Denmark, ^5^Médical Department, Region Sjælland, Nykoebing F., Denmark, ^6^Holbaek Hospital, Med.Afd. Gas. Amb., Holbaek, Denmark, ^7^Division of Gastroenterology, Department of Medicine, Holbaek Hospital, Holbaek, Denmark, ^8^Gastroenterology Department, University Hospital Vall de Hebron, Barcelona, Spain, ^9^Department of Gastroenterology, Lithuanian University of Health Sciences, Kaunas, Lithuania, ^10^Gastroenterology, Lithuanian University of Health Sciences, Kaunas, Lithuania, ^11^Gastroenterology, Hospital Universitari Mutua Terrassa, Terrassa, Spain, ^12^Gastroenterology and Endoscopy Unit, Fondazione IRCCS Ca' Granda Ospedale Maggiore Policlinico, Milan, Italy, ^13^Department of Gastroenterology, University Hospital "Tsaritsa Yoanna ‐ ISUL", Sofia, Bulgaria, ^14^CH de Marne La Vallée, Jossigny, France, ^15^Facharztzentrum Eppendorf, CED‐Schwerpunkt, Magen‐Darm‐Zentrumpraxis, University Professor of Internal Medicine, Hamburg, Germany, ^16^Department of Gastroenterology and Hepatology, Linköping University Hospital, Linköping, Sweden, ^17^Gastroenterology, Zealand University Hospital Køge, Køge, Denmark

**Contact E‐Mail Address**: verhaeghbas@gmail.com

**Introduction**: Knowledge on the long‐term disease course of microscopic colitis (MC) is unknown, and rests on retrospective studies. No predictive markers of disease course and disease activity have been identified. This 5‐year prospective follow‐up of a European incidence cohort aims to address these questions.

**Aims & Methods**: Incident cases of MC were prospectively included at 14 European centres. Patient characteristics and data on histology, disease activity, medical history, quality of life and treatment were registered. Patients were followed prospectively at 3, 6, 12 months and annually up to 5 years.

**Results**: Of the 501 incident cases, 62 declined participation and 17 had a colonoscopy for another reason than diarrhoea. Of the remaining 422 patients, 141 were lost to follow‐up (e.g. death, withdrawal), and 61 did not reach the 5‐year visit yet. Of the 220 cases with complete 5 year follow‐up; 73% female, mean age 63 years; 46% had collagenous colitis (CC), 41% lymphocytic colitis (LC) and 13% incomplete MC. At baseline, 82% reported urgency, 44% nightly defecation, 39% faecal incontinence, and 47% moderate to severe functional impairment. A majority of 67% had active disease according to Hjortswang criteria.^1^ Treatment with budesonide was initiated in 51%, 29% was treated with loperamide or bulging agents, and 20% was not treated.

During 5‐year follow‐up, only 5% of the patients followed a quiescent disease course, i.e., no disease activity or treatment ever since diagnosis. Sustained clinical remission was achieved in 21%, 35% and 55% after 1, 3 and 5 years, respectively. In contrast, 33% had a relapsing disease course, and 7% exhibited a chronic active course with continuous activity and/or treatment. The disease course did not differ between CC and LC. Stool frequency at baseline was not predictive.

A quiescent disease course since diagnosis or achieving sustained clinical remission during the first or second year, was associated with a 70% chance of being in clinical remission after 5 years (*p* < 0.001). In contrast, 57% of cases with a chronic active disease course in the first year had a chronic active or relapsing disease course throughout 5‐year follow‐up (*p* = 0.02).

Patient reported symptom severity (SHS item 1) and interference of bowel symptoms with daily life (SHS item 2), significantly improved from baseline to year 1 and year 5 (both *p* < 0.001). Cases with a quiescent course or achieved clinical remission after 5 years scored significantly better on SHS item 1 and 2, compared to those with relapsing or chronic disease activity (*p* < 0.001).

At 5‐year follow‐up, 53 patients (24%) were on budesonide use, of which 47 (89%) had a relapsing or chronic disease course. Of those, almost one third used budesonide on demand and 8% used it continuously since diagnosis. Bulging agents (15%) and loperamide (21%) were regularly applied after 5 years, mainly in relapsing cases. Of those in clinical remission, 17% used loperamide on demand. Treatment with thiopurines (<1%) and biologicals (<2%) during follow‐up was rare.

**Conclusion**: Five years after diagnosis, 40% of MC patients had a relapsing or chronic active disease course, resulting in an impaired quality of life. Disease activity at 1 year after diagnosis was predictive of activity after 5 years. Budesonide was the most frequently applied treatment, while use of immunosuppressants and biologicals was rare. Follow‐up of MC patients should reflect the recurrent course of disease, and physicians should inform them accordingly. Randomised trials on alternatives to longtime budesonide use are warranted.

**Reference**

1. Hjortswang, et al. IBD 2009;15:1875–1881.

**Disclosure**: The PRO‐MC project was primarily initiated after receiving the UEG LINK Award 2014

## Breakthroughs in screening and management of colorectal lesions: Late‐breaking abstracts Monday, 16 October 2023 10:00–11:00/A1

### LB06 COLD VS. HOT SNARE RESECTION OF NON‐PEDUNCULATED POLYPS ≥2CM IN THE COLORECTUM – FIRST RESULTS FROM THE PROSPECTIVE, RANDOMIZED, CONTROLLED, MULTICENTRIC CHRONICLE‐TRIAL

I. Steinbrück ^1^, A. Ebigbo^2^, A. Küllmer^3^, K. Kouladouros^4^, A. Meining^5^, T. Koenen^6^, V. Rempel^7^, A. Wannhoff^8^, S. Faiss^9^, O. Pech^10^, O. Möschler^11^, F. L. Dumoulin^12^, M. M. Kirstein^13^, T. von Hahn^14^, H.‐D. Allescher^15^, S. Gölder^16^, M. Götz^17^, S. Hollerbach^18^, B. Lewerenz^19^, A. Schmidt^20^, S. Nagl^2^, S. Belle^4^, M. Brand^5^, J. Tischendorf^6^, K. Caca^8^, M. Mende^9^, L. Pfeifer^10^, V. Weigart^15^, F. Wiedbrauck^18^, H. Messmann^2^, H.‐P. Allgaier^1^

^1^Department of Medicine and Gastroenterology, Evangelisches Diakoniekrankenhaus Freiburg, Freiburg, Germany, ^2^Department of Gastroenterology, Universitätsklinikum Augsburg, Augsburg, Germany, ^3^Department of Medicine II, Medical Center, University of Freiburg, Faculty of Medicine, Freiburg, Germany, ^4^Universitätsmedizin Mannheim, Zentrale Interdisziplinäre Endoskopie, Mannheim, Germany, ^5^Gastroenterology, University of Würzburg, Würzburg, Germany, ^6^Department of Internal Medicine and Gastroenterology, Rhein‐Maas Klinikum Würselen, Würselen, Germany, ^7^St. Anna Hospital, Klinik für Gastroenterologie, Herne, Germany, ^8^Department of Gastroenterology, RKH Klinikum Ludwigsburg, Ludwigsburg, Germany, ^9^Department of Gastroenterology, Sana Klinikum Lichtenberg, Berlin, Germany, ^10^Department of Gastroenterology and Endoscopy, Krankenhaus Barmherzige Brüder Regensburg, Regensburg, Germany, ^11^Department of Endoscopy and Ultrasound, Marienhospital Osnabrück, Osnabrück, Germany, ^12^Department of Medicine and Gastroenterology, Gemeinschaftskrankenhaus Bonn, Bonn, Germany, ^13^Department of Medicine I, University Hospital Lübeck, Lübeck, Germany, ^14^Department of Gastroenterology, Hepatology and Endoscopy, Asklepios Klinik Barmbek, Hamburg, Germany, ^15^Department of Gastroenterology, Klinikum Garmisch‐Partenkirchen, Garmisch‐Partenkirchen, Germany, ^16^Department of Internal Medicine I, Ostalb‐Klinikum Aalen, Aalen, Germany, ^17^Department of Gastroenterology/Oncology, Klinikum Sindelfingen‐Böblingen, Böblingen, Germany, ^18^Gastroenterology, AKH Celle, Celle, Germany, ^19^Department of Gastroenterology and Hepatology, Klinikum Traunstein, Traunstein, Germany, ^20^Department of Gastroenterology, Hepatology and Endocrinology, Robert‐Bosch‐Krankenhaus, Stuttgart, Germany

**Contact E‐Mail Address**: ingo.steinbrueck@diak‐fr.de

**Introduction**: For the removal of diminutive colorectal polyps <10 mm cold snare (CS) resection is standard of care. For polyps ≥20 mm endoscopic mucosal resection (EMR) with a hot snare (HS) is recommended, which is usually accomplished in piece meal technique.^1^ Recently, a retrospective trial showed a favorable safety profile and a comparable recurrence rate for CS‐EMR in comparison to HS‐EMR for sessile serrated adenomas (SSA) ≥20 mm^2^. We investigated if CS‐EMR is superior to HS‐EMR for the removal of larger, non‐pedunculated, colorectal polyps.

**Aims & Methods**: In this prospective, randomized, controlled trial non‐pedunculated colorectal polyps ≥20 mm were randomly assigned to CS‐EMR or HS‐EMR. Primary endpoint was major complication (=perforation or clinically significant post‐endoscopic bleeding [CSPEB]), secondary endpoints were intraprocedural bleeding (IPB), postpolypectomy syndrome (PPS), technical success, resection speed and recurrence rate after 4 months.

**Results**: 394 polyps were finally enrolled for intention‐to‐treat analysis. Rates of major complication were significantly lower in the CS‐EMR group with 1.0% versus 8.0% (*p* = 0.001) including rates of perforation of 0% versus 4.0% (*p* = 0.007) and rates of CSPEB of 1.0% versus 4.5% (*p* = 0.038). Independent predictor for major complication in uni‐/multivariable regression analysis was polyp size (OR 1.10).

Rates of IPB were 14.0% for CS‐EMR and 22.9% for HS‐EMR (*p* = 0.023), rates of PPS 3.1% and 4.5% (*p* = 0.478). Rates of technical success were 92.2% in CS‐EMR group and 97.5% in HS‐EMR group (*p* = 0.017), resection speed was 22.61 (±16.62) cm^2^/h and 21.71 (±19.27) cm^2^/h (*p* = 0.248) and recurrence rates 24.8% for CS‐EMR and 15.0% for HS‐EMR (*p* = 0.037). In subgroup analysis a significant difference was observed for the recurrence rate of Laterally spreading tumors (LST) nodular‐mixed type (43.8% vs. 16.7%, *p* = 0.014) but not for suspected SSA (5.9% vs. 5.3%, *p* = 1), LST non‐granular type (11.5% vs. 11.1%, *p* = 1) and LST granular‐type homogenous (33.3% vs. 23.9%, *p* = 0.320).

**Conclusion**: Safety of CS‐EMR is superior to HS‐EMR for the removal of large, non‐pedunculated colorectal polyps with an almost complete elimination of major complications. As general recurrence rate is higher after CS‐EMR careful selection of the target lesion should be made. CS‐EMR should be considered as new standard of care for suspected SSA, LST non‐granular type with no macroscopic signs of malignancy and selected LST granular‐type homogenous ≥20 mm.

**References**

1. Ferlitsch M, Moss A, Hassan C, Bhandari P, Dumonceau JM, Paspatis G, et al. Colorectal polypectomy and endoscopic mucosal resection (EMR): European Society of Gastrointestinal Endoscopy (ESGE) clinical guideline. *Endoscopy* 2017;49(03):270–297.
2. Hattem A, Shahidi N, Vosko S, Hartley I, Britto K, Sidhu M, et al. Piecemeal cold snare polypectomy versus conventional endoscopic mucosal resection for large sessile serrated lesions: a retrospective comparison across two successive periods. *Gut* 2021;70(9):1691–1697.

**Disclosure**: Ingo Steinbrück: lecture fees and travel grants from Olympus and Falk Pharma.

Helmut Messmann: none.

Alanna Ebigbo: lecture fees from Olympus Medical, FujiFilm, Pentax, Ambu, Falk Pharma and Medtronic.

Sandra Nagl: lecture fees from Falk Pharma, Pfizer and Sanofi.

Armin Küllmer: lecture fees from Ovesco Endoscopy AG and Falk Pharma and consulting fees from KLS Martin Group, Tuttlingen, Germany.

Sebastian Belle: none, Konstantinos Kouladouros: none.

Alexander Meining: consultancy from Ovesco Endoscopy AG and Olympus Medical.

Markus Brand: none, Jens Tischendorf: none, Teresa Koenen: none.

Viktor Rempel: lecture fees and travel grants from Olympus and lecture fees from Microtec.

Andreas Wannhoff: none, Carel Caca: none.

Siegbert Faiss: lecture fees and consulting fees from Olympus and Ovesco Endoscopy AG.

Matthias Mende: none

Oliver Pech: lecture fees from Medtronic, Aohua, AbbVie, Falk Pharma and Boston Scientific

Lukas Pfeifer: none.

Oliver Möschler: lecture fees from Olympus Medical and FujiFilm.

Franz‐Ludwig Dumoulin: lecture fees and travel grants from Olympus.

Martha Kirstein: none.

Thomas von Hahn: lecture and consulting fees from Olympus.

Hans‐Dieter Allescher: none.

Stefan Gölder: Lecture fees from Astra Zeneca, Falk Pharma, Pfizer and Apollo Endosurgery, funding of educational events from Advanz, Abbvie, B. Braun, Fresenius, Lilly, MSD, Janssen, Roche and Takeda.

Martin Götz: consulting fees from Abbvie, Janssen and Galapagos, member of advisory board of Boehringer Ingelheim and Alexion, lecture fees from Abbvie, Takeda, Pentax, MSD and DGVS.

Stephan Hollerbach: none.

Felix Wiedbrauck: none.

Björn Lewerenz: none.

Arthur Schmidt: lecture fees from Falk Pharma and Olympus Medical.

Hans‐Peter Allgaier: lecture fees and travel grants from Olympus.

## LB07 | RECURRENCE IN LARGE NON‐PEDUNCULATED COLONIC LESIONS IS SIGNIFICANTLY HIGHER AFTER COLD SNARE ENDOSCOPIC MUCOSAL RESECTION THAN AFTER THE STANDARD TECHNIQUE. RESULTS OF A RANDOMIZED CONTROLLED TRIAL

Ó. Nogales ^1^, C. Carbonell Blanco^1^, M. Pellisé^2^, J. F. Martínez Sempere^3^, F. Riu Pons^4^, C. Mangas‐Sanjuan^3^, M. Daca Alvarez^5^, H. Uchima^6^, J. Aranda Hernández^1^, A. Alvarez Delgado^7^, E. Albéniz‐Arbizu^8^, GSEED de resección endoscópica (RMEFRÍA.2019 group)

^1^Gastroenterology and Hepatology, Hospital General Universitario Gregorio Marañón, Madrid, Spain, ^2^Gastroenterology, Hospital Clinic de Barcelona, Institut d'Investigacions Biomediques August Pi I Sunyer (IDIBAPS), Barcelona, Spain, ^3^Gastroenterology, Hospital Universitario de Alicante Dr. Balmis, Alicante, Spain, ^4^Gastroenterology Department, Hospital del Mar, Barcelona, Spain, ^5^Gastroenterology, Hospital Clinic, Barcelona, Spain, ^6^Gastroenterology (GI Endoscopy Unit), Hospital Germans Trias i Pujol, Barcelona, Spain, ^7^Gastroenterology, Hospital Universitario de Salamanca, Salamanca, Spain, ^8^Servicio Aparato Digestivo, Hospital Universitario de Navarra, Pamplona, Spain

**Contact E‐Mail Address**: oscarnogalesrincon@gmail.com

**Introduction**: Cold snare EMR (CS‐SEMR) in large non‐pedunculated colonic lesions is an alternative to the standard procedure (C‐EMR), with a better theoretical safety profile. Robust scientific evidence on its efficacy is not available.

**Aims & Methods**: Objectives: Primary: to compare the efficacy between the two techniques, measured as the absence of recurrence at 6 months. Secondary: to compare safety profile and technical aspects.

This is a randomized controlled trial (1:1), multicenter, non‐blinded, of consecutive non‐pedunculated lesions with adenoma or serrated histology, homogeneous type, with size ≥20 mm.

**Results**: 229 patients, mean age 68 years, male 56.7% (*N* = 130), were randomized to CS‐EMR (*N* = 115) or C‐EMR (*N* = 114). Overall, 49% were homogeneous granular LST lesions, predominantly adenomas (74.2%); median size 25 mm. No differences between groups in baseline or lesion characteristics were found. The recurrence rate at first surveillance colonoscopy (*N* = 215) was significantly higher in the CS‐EMR group versus C‐EMR group: 33.6 versus 16.7% (*p* = 0.007), and 35.1 versus 15.2% (*p* = 0.002) in ITT and PP analysis, respectively. En bloc and R0 resection rates were higher in C‐EMR versus CS‐EMR: 23.7% versus 1.7% *p* = 0.001, and 21.1% versus 1.7% *p* = 0.001, respectively. There was no difference in the rate of complications: delayed bleeding (CS‐EMR 2.6% vs. C‐EMR 3.5% *p* = 0.722), perforation (0% in both groups) and post‐polypectomy syndrome (CS‐EMR 0.9% vs. C‐EMR 1.8% *p* = 0.622). No differences were found in resection time either: CS‐EMR 28.23 min versus C‐EMR 22.30 min, *p* = 0.297. The median number of fragments was higher in CS‐EMR (6) versus C‐EMR (3) *p* = 0.001, while the use of clips was higher in C‐EMR (2.21) versus CS‐EMR (1.30) *p* = 0.001.

**Conclusion**: The recurrence rate is significantly higher after CS‐EMR compared to the standard technique. There is a trend toward fewer adverse effects in CS‐EMR, without statistical significance in our study.

**Disclosure**: Nothing to disclose.

## LB08 | RISK STRATIFICATION IN COLORECTAL CANCER SCREENING: INTERVAL CANCER RISK AFTER THE UPPER AGE LIMIT OF SCREENING HAS BEEN REACHED

B. van Stigt ^1^, I. Lansdorp‐Vogelaar^1^, E. Toes‐Zoutendijk^1^

^1^Public Health, University Medical Center Rotterdam, Rotterdam, Netherlands

**Contact E‐Mail Address**: b.vanstigt@erasmusmc.nl

**Introduction**: Colorectal cancer (CRC) screening programmes have been shown effective in reducing CRC incidence and mortality. Risk stratification offers opportunities for further optimisation of screening programmes by better balancing the harms and benefits of screening. To this day, nearly all population screening programmes only differentiate their screening approach by age, resulting in a fixed upper age limit of screening. While further risk stratification based on prior faecal haemoglobin (f‐Hb) concentration seems promising, studies are mainly limited to the target population of screening. Risk‐stratified screening beyond the upper age limit might however be beneficial as well considering the increasing life expectancy and the benefit‐risk ratio of screening widely varying amongst elderly.

**Aims & Methods**: This study assessed interval colorectal cancer (IC) risk in individuals that have reached the upper age limit of screening (75 years), and whether this risk differs by the f‐Hb concentration of the last faecal immunochemical test (FIT). Individuals with a negative FIT (<47 μg Hb/g faeces) in their final CRC screening round were selected in the national screening database and linked to the Dutch cancer registry to check for a CRC diagnosis within 24 months of the negative FIT. IC risk was compared between different risk groups (sex, prior f‐Hb concentration and screening round) and tested with a Chi‐squared test. Associations between IC and explanatory factors were assessed using survival and Cox proportional hazards analyses. Factors associated with CRC stage were identified using a multivariable logistic regression analysis.

**Results**: A total of 311,655 individuals with a complete follow‐up (24 months) after a negative FIT were included. Within this population, the overall IC risk was 21.6 per 10,000 individuals with a negative FIT. Individuals with detectable f‐Hb (>0 µg Hb/g faeces) in the final screening round developed IC more often than those without (HR 4.85, 95% CI: 4.18–5.63) (Table 1). Besides, they were also more likely to be diagnosed at an advanced rather than early stage of the disease (OR 1.45, 95% CI: 1.05–2.00). Individuals who had participated in three or four screening rounds had a lower IC risk than those who had participated once (HR 0.75, 95% CI: 0.56–0.99). Screening round was not associated with stage distribution and sex was not associated with IC nor stage distribution.

**TABLE 1:** Overview of IC risk, including results of a Cox regression analysis with IC as a dependent variable and sex, screening round and prior f‐Hb concentration as independent variables.

Variable	Category	IC risk (per 10,000 negative FITs)	HR (95% CI)
Sex	Men	23.4	Ref
	Women	20.0	0.90 (0.78–1.04)
Screening round	1	22.8	Ref
	2	17.5	0.95 (0.78–1.18)
	3 or 4	15.2	0.75 (0.56–0.99)
Prior f‐Hb concentration	0 μg Hb/g faeces	13.8	Ref
	>0 μg Hb/g faeces	65.6	4.85 (4.18–5.63)

Abbreviations: CI, confidence interval; f‐Hb, faecal haemoglobin; FIT, faecal immunochemical test; IC, interval colorectal cancer; Ref, reference.

**Conclusion**: Our results indicate that a risk‐stratified upper age limit in CRC screening based on prior f‐Hb concentration may prevent CRC cases and CRC‐related morbidity by early detection. While individuals with detectable f‐Hb in their final FIT may benefit from additional screening, screening of the elderly is not without risks. Follow‐up studies should therefore assess the benefit‐risk ratio of a risk‐stratified upper age limit and identify the optimal screening scenario.

**Disclosure**: Nothing to disclose.

## LB09 | RESULTS AND QUALITY PERFORMANCE OF A STATE‐WIDE COLORECTAL CANCER SCREENING INITIATIVE FOR 10,000 ELIGIBLE EMPLOYEES OF A HEALTH‐CARE PROVIDER

J. Prosenz ^1,2,3^, Z. A. Österreicher^1,2^, F. Koutny^1,2,3^, A. Asaturi^2^, M. Birkl^1,2^, R. Hanke^1^, E. Aigner^4^, A. Maieron^1,2,3^

^1^Karl Landsteiner University of Health Sciences, Krems, Austria, ^2^Internal Medicine 2 Gastroenterology & Hepatology, University Hospital St. Pölten, St. Pölten, Austria, ^3^Paracelsus Medical University, Salzburg, Austria, ^4^First Department of Medicine, Paracelsus University Salzburg, Salzburg, Austria

**Contact E‐Mail Address**: julian.prosenz@stpoelten.lknoe.at

**Introduction**: Existence of structured colorectal cancer (CRC) screening programs across Europe is inconsistent, moreover, participation rates vary greatly. Similarly, such a program as well as mandatory colonoscopy quality assurance are lacking in Austria. Loco‐regional, corporate preventive health initiatives might provide a more direct, effective CRC screening and increase uptake.

**Aims & Methods**: A state‐wide CRC screening of a health care provider with approximately 28,000 employees was planned. We aimed to assess the performance in a real‐world setting measured by acceptance rates of a defined target population and quality of colonoscopies performed by unselected endoscopists. All employees ≥50 years were invited (personal invitation letter and promotion at the facilities). Invitees received a stool‐based test (FIT and M2PK). Stool tests could be performed at home, dropped at the workplace, and were collected and analyzed at a central facility. All persons with positive tests were called and directly offered a colonoscopy near their work‐place/home within 3 weeks. Persons without a colonoscopy registered in records were called up again and received reminders via E‐mail. Colonoscopy and histology reports as well as basic demographic data were collected (IRB vote GS1‐EK‐4/735‐2021).

**Results**: Of 10,239 eligible employees (2706 males, 7533 females), 2390 (23%) (plus 609 <50 years) age 52.3 (SD 5.4 years) participated in the stool‐based screening (18% of males, 25% of females). Of 3063 overall tests, 747 (24%) were positive – more likely in males (29%) than females (23%) (RR 1.25 [95% CI 1.07–1.46]). Of 747 positive tests, a minority was FIT‐ (*n* = 179, 24%), while the majority was M2PK‐only (*n* = 568, 76%) positive. A colonoscopy was offered to 747 persons, 117 (16%) refused, 36 (5%) are still undecided, and 593 (79%) initially accepted. The acceptance rate was lower in men (77%) than women (80%). The follow‐up rate of 616 persons who accepted or eventually underwent colonoscopy (some after initial refusal) was 84% (*n* = 517 and 1 virtual colonoscopy). Of the 517 colonoscopies, 177 (34%) were performed at a hospital and 340 (66%) by office‐based physicians, 304 (59%) were performed by internists/gastroenterologists and 213 (41%) by surgeons. The histological polyp detection rate (PDR) was 32% (*n* = 167). The adenoma detection rate (ADR) was 20.5% (FIT‐positive ADR 25.6%) and varied substantially. The ADR was 18.5% among office‐based endoscopists and 24.3% in‐hospital – however, 15% when excluding endoscopies from the University Hospital of St Pölten (study/reference center, ADR of 36%). No high‐grade dysplasias or CRC were detected. The caecum intubation rate was (reportedly) 97%, yet the complete colonoscopy rate (caecum reached, adequate visualization reported) was 84.7%. Bowl preparation was adequate in 88%, a BBPS was reported only in 49%, and merely 10% reported withdrawal times. Polyp sizes were noted in 76% (or 48% if “polyp bud” was not counted), a PARIS or NICE classification was provided in 27%. Of 140 colonoscopies with polyps ≤10 mm, 54% were removed by forceps, 29% by cold snare, and 6% by hot snare. Only 54% of polyps >10 mm were removed during initial colonoscopy. No major complications were reported.

**Conclusion**: Acceptance rates of CRC screening even among health care(‐associated) persons are low and lowest among the highest risk group – men >50a. New strategies to reach high risk populations, possibly adapted to individual groups, must be devised. An ADR of 15%–36% among unselected endoscopists in a pre‐screened population denotes poor quality, corroborated by low adherence to quality measures. These data support the drive and underscore the urgent need for rigorous quality control in CRC screening colonoscopy.

**References**

1. “Colorectal Screening Across Europe” UEG. 2023. Available from: https://ueg.eu/
2. Kaminski MF, et al. Performance measures for lower gastrointestinal endoscopy: a European Society of Gastrointestinal Endoscopy (ESGE) Quality Improvement Initiative. *Endoscopy* 2017;49(4):378–397. 10.1055/s‐0043‐103411. Epub 2017 Mar 7.

**Disclosure**: Tillotts Pharma AG ‐ Travel support UEG Week 2023.

## LB10 | IMPACT OF ANNUAL CASE VOLUME ON COLORECTAL ENDOSCOPIC SUBMUCOSAL DISSECTION PROCEDURAL OUTCOMES AND SAFETY IN A LARGE MULTICENTRIC PROSPECTIVE COHORT STUDY

L. Alfarone ^1^, M. Schaefer^2^, T. Wallenhorst^3^, V. Lepilliez^4^, T. Degand^5^, Y. Le Baleur^6^, P. Leclercq^7^, A. Berger^8^, E. Chabrun^8^, B. Brieau^9^, M. Barret^9^, G. Rahmi^10^, R. Legros^11^, J. Rivory^12^, S. Leblanc^4^, G. Vanbiervliet^13^, Z. Jean‐Baptiste^7^, J. Albouys^11^, G. Perrod^10^, C. Yzet^12^, H. Lepetit^11^, A. Belle^9^, S. Chaussade^9^, F. Rostain^12^, M. Dahan^8^, A. Lupu^12^, M. Pioche^12^, J. Jacques^11^

^1^Gastroenterology and Endoscopy, Humanitas Research Hospital, Milan, Italy, ^2^Hepato‐Gastroenterologie, Centre Hospitalier Régional Universitaire de Nancy, Nancy, France, ^3^Endoscopy Unit, CHU Pontchaillou, Rennes, France, ^4^Endoscopy and Gastroenterology, Hôpital Privé Jean Mermoz, Lyon, France, ^5^Gastroenterology, CHU de Dijon, Dijon Cedex, France, ^6^Groupe Hospitalier Paris Saint Joseph, Paris, France, ^7^Gastroenterology, CHU ST, Liége, Belgium, ^8^Endoscopy, Hôpital Haut‐Levêque, CHU Bordeaux, Bordeaux, France, ^9^Service de Gastro‐Entérologie, Cochin University Hospital, Paris, France, ^10^Department de Gastroenterologie, Hôpital Européen Georges Pompidou, Paris, France, ^11^CHU Limoges, Limoges, France, ^12^Hopital Edouard Herriot, Lyon, France, ^13^Gastroenterology, CHU, Hôpital L'Archet 2, Nice, France

**Contact E‐Mail Address**: ludovico.alfarone@humanitas.it

**Introduction**: The adoption of colorectal endoscopic submucosal dissection (ESD) as the main treatment for large colorectal lesions is still limited in the Western world. Through the implementation of novel strategies and training programs, ESD has been proved more effective than piecemeal endoscopic mucosal resection (EMR) for large colorectal lesions, without a significantly higher risk of adverse events, according to recent high‐quality data. Reproducibility outside experts centers has been questioned.

**Aims & Methods**: Therefore, we evaluated results according to volume case load per year in a large multicentric prospective cohort of colorectal ESDs. Patients referred for colorectal ESD were consecutively included in this multicentric prospective cohort (FECCO NCT04592003) between 09/2019 and 09/2022. Participating centers were classified into low‐volume (LV) (<50 ESDs/year), middle‐volume (MV) (50–100 ESDs/year), and high‐volume (HV) centers (>100 ESDs/year). Efficiency was assessed in terms of en bloc, R0, and curative resection rates and dissection speed, while rates of complications and need for surgery were the main safety outcomes. Both univariate and multivariate analyses were performed for our analysis.

**Results**: 3770 colorectal ESDs performed in 13 centers by 29 operators were included. 62.4% of the lesions were located in the colon and 37.6% in the rectum. Median large and small lesion diameters were 50 and 40 mm, respectively. HV group performed significantly more colonic cases (68.6%) than MV and LV center groups (61.6% and 40.5%, respectively) (*p* < 0.0001).

Lesions were significantly larger in HV and MV centers (50 × 40 mm for both) in comparison to LV centers (45 × 35 mm) (*p* < 0.0001). The overall en bloc resection rate was 95.2%; HV centers achieved a significantly greater rate (96.3%) than MV and LV centers (92.9% and 93.9%, respectively) (*p* < 0.01). Pooled R0 resection rate was 87.4%; it was better in HV centers than in MV and LV centers (88.7% vs. 83.8% and 86.8%, respectively). Similarly, the curative resection rate, being overall 83.2%, was lower in MV centers (79%) in comparison to HV and LV groups (84.7% [*p* < 0.01] and 82.5% [*p* = 0.10], respectively).

The median duration of procedure was 57 min (IQR 35–90) and was shorter in HV centers (48 min, IQR 30–80) than LV and MV group (75 min, IQR 45–120 and 70 min, IQR 42.8–120.5, respectively) (*p* < 0.0001). Dissection speed was 28.7 mm^2^/min (IQR 16.8–45.8), being greater in HV centers (34.8 mm^2^/min, IQR 21.3–51.4) and lower in MV (22.9 mm^2^/min, IQR 14.7–34.5) (*p* < 0.0001) and LV centers (18.1 mm^2^/min, IQR 10.5–28.6) (*p* < 0.0001).

All three groups exceed the recommended thresholds of acceptability with satisfactory quickness. Globally, delayed bleeding and surgery due to complications occurred in 5.4% and 0.8% of cases, respectively, with no significant difference between the three groups. Perforation rate, being overall 9%, was higher in MV centers (11.1%) than LV and HV groups (7.5% [*p* = 0.02] and 8.7% [*p* = 0.06], respectively).

Multivariate analysis found that size >50 mm, poor maneuverability, recurrent and appendiceal lesions, but not volume of the center were risk factors for both R1 resection (*p* < 0.001) and perforation (*p* < 0.001) suggesting that differences of outcomes can be explained by lesion characteristics.

**Table:** 

Data	Full cohort	Low‐volume center	Middle‐volume center	High‐volume center	*p*‐value (full cohort)	*p*‐value (low vs. middle)	*p*‐value (low vs. high)	*p*‐value (middle vs. high)
Longest diameter, median (IQR), mm	50 (37; 65)	45 (32; 60)	50 (40; 70)	50 (39.8; 65)	**<0.0001**	**<0.0001**	**<0.0001**	0.290
Rectal/colonic lesions, *n* (%)	1417/2353 (37.6/62.4)	383/261 (59.5/40.5)	285/457 (38.4/61.6)	749/1635 (31.4/68.6)	**<0.0001**	**<0.0001**	**<0.0001**	**<0.0001**
Dissection speed, median (IQR), mm^2^/min	28.7 (16.8; 45.8)	18.1 (10.5; 28.6)	22.9 (14.7; 34.5)	34.8 (21.3; 51.4)	**<0.0001**	**<0.0001**	**<0.0001**	**<0.0001**
En bloc resection, *n* (%)	3590 (95.2)	605 (93.9)	689 (92.9)	2296 (96.3)	**<0.0001**	0.418	**0008**	**<0.0001**
R0, *n* (%)	3293 (87.4)	559 (86.8)	622 (83.8)	2112 (88.7)	**0.002**	0.120	0.192	**<0.0001**
Curative resection, *n* (%)	3134 (83.2)	531 (82.5)	586 (79)	2017 (84.7)	**0.001**	0.103	0.170	**<0.0001**
Perforation, *n* (%)	335 (9)	48 (7.5)	82 (11.1)	205 (8.7)	0.054	**0.022**	0.301	0.059
Delayed bleeding, *n* (%)	199 (5.4)	29 (4.5)	47 (6.3)	123 (5.4)	0.328	0.139	0.382	0.329
Secondary surgery due to complications, *n* (%)	30 (0.8)	7 (1.1)	6 (0.8)	17 (0.7)	0.497	0.845	0.556	0.345

**Conclusion**: This study shows that, with appropriate training and organization, colorectal ESD can be widely implemented in the West with good outcomes even in not expert contests. Thus, under these conditions, ESD should be adopted as the first‐line treatment for large colorectal lesions also in the Western world. Nevertheless, challenging lesions should still be referred to expert centers.

**Disclosure**: Nothing to disclose.

## Advances in endoscopy: Late‐breaking abstracts Tuesday, 17 October 2023 08:30–09:30/D5

### LB11 EPINEPHRINE ADDED SOLUTION SIGNIFICANTLY REDUCED PROCEDURAL TIME DURING GASTRIC ENDOSCOPIC SUBMUCOSAL DISSECTION (ESD) – RESULTS FROM AN INTERNATIONAL DOUBLE BLINDED MULTI‐CENTRE RANDOMIZED CONTROLLED TRIAL

H. C. Yip ^1^, N. Uedo^2^, H. Iwagami^3^, K. Otsu^4^, K. Yao^4^, T. Akamatsu^3^, H. Doyama^5^, H. Machida^6^, K. Jung^7^, L. H.‐S. Lau^8^, K. Uchita^9^, T. Kawamura^10^, K. Takizawa^11^, Y. Kitamura^12^, J. Li^13^, T.‐L. Ang^13^, P. W. Y. Chiu^1^

^1^Department of Surgery, The Chinese University of Hong Kong, Hong Kong, Hong Kong, ^2^Department of Gastrointestinal Oncology, Osaka International Cancer Institute, Osaka, Japan, ^3^Gastroenterology and Hepatology, Japan Red Cross Wakayama Medical Center, Wakayama‐city, Japan, ^4^Department of Endoscopy, Fukuoka University Chikushi Hsopital, Chikushino, Japan, ^5^Department of Gastroenterology, Ishikawa Prefectural Central Hospital, Kanazawa, Japan, ^6^Department of Internal Medicine, Machida Gastrointestinal Hospital, Osaka, Japan, ^7^College of Medicine, Kosin University Gospel Hospital, Busan, South Korea, ^8^Division of Gastroenterology, Department of Medicine and Therapeutics, The Chinese University of Hong Kong, Hong Kong, Hong Kong, ^9^Department of Gastroenterology, Kochi Red Cross Hospital, Kochi, Japan, ^10^Department of Gastroenterology, Kyoto Second Red Cross Hospital, Kyoto, Japan, ^11^Department of Gastroenterology and Endoscopy, Koyukai Shin‐Sapporo Hospital, Sapporo, Japan, ^12^Department of Gastroenterology and Hepatology, Center for Digestive and Liver Diseases, Nara City Hospital, Nara City, Japan, ^13^Gastroenterology and Hepatology, Changi General Hospital, Singapore, Singapore

**Contact E‐Mail Address**: hcyip@surgery.cuhk.edu.hk

**Introduction**: Endoscopic submucosal dissection (ESD) has become a standard therapy for early gastric cancer. The type of submucosal injection solution used during gastric ESD varies among different endoscopists. The use of epinephrine added solution may facilitate the procedure by inducing vasoconstriction, thus minimizing intra‐operative haemorrhage and allowing clearer dissection plane. The efficacy of epinephrine added solution was previously reported in a single center retrospective study, demonstrating reduction in procedure time with the addition of epinephrine.^1^ In this prospective double blinded international randomized study, we aim to investigate whether using epinephrine added solution, could significantly reduce procedure time and the number of intra‐operative bleeding episodes.

**Aims & Methods**: This trial was conducted in 12 institutions across four countries. Adult patients with biopsy confirmed gastric mucosal neoplasia undergoing ESD would be recruited. Patients with multiple or anastomotic lesions, on dual antiplatelet agent, warfarin and other direct oral anticoagulants would be excluded. They would be randomized to epinephrine group which 0.2 mL 1:1000 epinephrine diluted into each 20 mL of the original injection solution mixture, and control group which no epinephrine would be added. Randomization was stratified with lesion location (upper/middle vs. lower), size () and institution. The primary outcome was procedure time defined from the beginning of mucosal incision to the completion of resection. Secondary outcomes include intra‐operative haemorrhage requiring use of hemostatic forceps, peri‐procedure blood pressure, pulse and post‐procedure adverse events. Patients, endoscopists and assessors were blinded to the treatment allocation.

**Results**: From March 2020 to January 2023, 800 patients were recruited with 774 included in the final intention‐to‐treat analysis. Three hundred and eighty‐six (49.9%) and 388 (50.1%) patients were randomized to epinephrine and control group respectively. One hundred thirty‐three (17.2%), 286 (37.0%), 355 (45.9%) of the lesions were located in the upper, middle and lower part of the stomach respectively, and majority (87.3%) of the lesions were 46.21 versus 68.06 45.37 min, adjusted mean difference −6.977 min, [−12.756, −1.198], *p* = 0.01. The number of intra‐operative bleeding episodes requiring use of hemostatic forceps was also significantly lower in the epinephrine group (mean count 1.76 vs. 3.02, adjusted OR 0.59 [0.54–0.65], *p* < 0.001). No significant difference was identified in post‐procedural adverse event between the two groups.

**Table:** 

	Treatment (*n* = 386)	Control (*n* = 388)	Odds/rate ratio/mean diff. [95% CIs] (adjusted)	*p*‐value
Location: Upper & middle	191 (49.5%)	228 (58.8%)		
Location: Lower	195 (50.5%)	160 (41.2%)		
Anterior wall	54 (14.0)	65 (16.8)		
Posterior wall	75 (19.4)	68 (17.5)		
Lesser curvature	167 (43.3)	182 (46.9)		
Greater curvature	90 (23.3)	73 (18.8)		
Endoscopic lesion size>2 cm	56 (14.5%)	42 (10.8%)		
≤2 cm	330 (85.5%)	346 (89.2%)		
Procedure time (min)	59.96 (46.21)	68.06 (45.37)	−6.977 [‐12.756, −1.198]	0.018
Number of intra‐procedural haemorrhage	1.76	3.02	0.59 [0.54, 0.65]	<0.001
Delayed hemorrhage	21 (5.5%)	11 (2.8%)	0.53 (0.25, 1.12)	0.096
Perforation	10 (2.6%)	6 (1.5%)	0.54 (0.19, 1.52)	0.245

**Conclusion**: Conclusion.

The use of epinephrine added solution significantly shortens the procedure time and reduces intraoperative bleeding events during gastric ESD.

**Reference**

1. Inoue S, Uedo N, Tabuchi T, Nakagawa K, Ohmori M, Iwagami H, et al. Usefulness of epinephrine‐added injection solution to reduce procedure time for gastric endoscopic submucosal dissection. *Endosc Int Open*. 2020;8(8):E1044–E1051. 10.1055/a‐1192‐4202. Epub 2020 Jul 21. PMID: 32743058; PMCID: PMC7373655

**Disclosure**: This study was supported by Asian Endoscopic Research Forum (AERF).

## LB12 | ENDOSCOPIC SELF‐EXPANDABLE METAL STENT VERSUS ENDOSCOPY VACUUM THERAPY FOR TRAUMATIC OESOPHAGEAL PERFORATIONS: THE FORTAL RANDOMISED DOUBLE‐BLIND CONTROLLED COMPARATIVE EFFECTIVENESS TRIAL

A. Terceiro de Oliveira ^1^, A. Cyntia Lima Fonseca Rodrigues^2^, J. W. da Cunha Parente Júnior^3^, J. R. Lima Herculano Junior^1^, J. Borges e Silva Ribeiro^4^, O. Gomes Ripardo de Azevedo^5^, P. R. Cavalcante de Vasconcelos^5^

^1^Department of Digestive Endoscopy, General Hospital of Fortaleza, Fortaleza, Brazil, ^2^Department of Medicine, Positivo University, Curitiba, Brazil, ^3^Department of Digestive Endoscopy, Instituto Dr. Jose Frota, Fortaleza, Brazil, ^4^Department of Digestive Endoscopy, Federal University of Piaui, Teresina, Brazil, ^5^Department of Surgery, Federal University of Ceara, Fortaleza, Brazil

**Contact E‐Mail Address**: aleterceiro@yahoo.com.br

**Introduction**: Oesophageal injuries and perforations are transmural ruptures of the oesophagus that subsequently lead to leakage of intraluminal contents into the surrounding mediastinum. This causes local inflammation, a systemic inflammatory response, and eventually the development of sepsis, resulting in significant morbidity and mortality.

**Aims & Methods**: This study aimed to compare oesophageal self‐expandable metal stent (E‐SEMS) with endoscopic vacuum therapy (EVT) in oesophageal perforations (EP) treatment. We randomly evaluated 30 patients with traumatic EP. The E‐SEMS and EVT groups were assessed for treatment duration, duration of hospitalisation, LOS (length of stay), clinical outcomes, and treatment success.

**Results**: In both groups, the most common cause of injury was a foreign body 63.3% (19/30), followed by gunshot wounds 30.0% (9/30) and stab wounds 6.7% (2/30). The treatment duration for E‐SEMS (45.8 ± 12.9) was significantly longer than EVT (24.42 ± 13.21), *p* < 0.005. Evaluating the clinical results, E‐SEMS demonstrated a 93.75% (15/16) discharge rate compared to EVT which showed 71.4% (10/14). Deaths occurred in the E‐SEMS and EVT groups at rates of 6.3% (01/16) and 28.6% (04/14) respectively, *p* = 0.1571. Treatment success showed no significant difference between E‐SEMS 93.7% (15/16) and EVT 78.6% (11/14), *p* = 0.3155. The costs were calculated at €19,266.08 ± 10,925.00 for E‐SEMS and €38,453.17 ± 23,840.40 for EVT, respectively.

**Conclusion**: E‐SEMS had a longer treatment duration compared to EVT. Both therapies showed no significant difference in clinical outcome and treatment success. The overall cost was lower for E‐SEMS compared to EVT.

**Disclosure**: Nothing to disclose.

## LB13 | SERUM PEPSINOGEN 1 AND PESINOGEN1 TO PEPSINOGEN 2 RATIO LEVEL AS A SCREENING FOR ATROPHIC GASTRITIS AND GASTRIC CANCER

A. M. Shamseya ^1^, A. Elbanna^1^, W. Hussein^1^, M. Abozamel^1^

^1^Internal Medicine, Gastroenterology Unit, Faculty of Medicine, University of Alexandria, Alexandria, Egypt

**Contact E‐Mail Address**: Dr.ayman1977@gmail.com

**Introduction**: Gastric cancer (GC) is the fourth most prevalent cancer‐related death factor. It has a great prognosis and is treatable when diagnosed at an early stage or early lesions that are likely to be precancerous, such as atrophic gastritis (AG). Pepsinogen is the most reliable non‐invasive biomarker for the diagnosis of severe AG or GC. However, its application is still controversial, and it is advised to utilize it carefully and with caution since the populations' cut‐off values vary.

**Aims & Methods**: To evaluate pepsinogen 1 (Pg1) level in serum and pepsinogen 1 to pepsinogen 2 ratio (Pg1/Pg2) in patients with atrophic gastritis (AG) and gastric cancer (GC), to detect sensitivity and specificity of these markers and evaluate their value as a future tumor marker. 600 patients diagnosed with either atrophic gastritis (group 1 of 300 patients) or gastric adenocarcinoma (group 2 of 300 patients) and 100 healthy volunteers as a control group were enlisted. A clinical and endoscopic examination was used to establish diagnosis, and gastric biopsies were examined histologically to confirm it. Serum Pg1 and Pg2 and Pg1/Pg2 ratio were estimated by Enzyme‐linked immunosorbent assay (ELISA).

**Results**: Serum Pg1 and Pg1/Pg2 showed comparable values for both patient's groups (*p* = 0.098, 0.976, respectively), however, compared to healthy controls, there was a statistically significant difference (*p* < 0.001). The optimum cut‐off values for diagnosing AG or GC were Pg1 18.96 and Pg 1/2 2.25; these values produced a sensitivity (94%), specificity (88.4%), positive predictive value (PPV) of 100%, and negative predictive value (NPV) of 100%.

**Conclusion**: Serum Pg1 and Pg1/Pg2 ratio demonstrated a very high diagnostic value for diagnosis of AG and GC and is a promising future tumor marker.

**References**

1. Machlowska J, Baj J, Sitarz M, Maciejewski R, Sitarz R. Gastric cancer: epidemiology, risk factors, classification, genomic characteristics and treatment strategies. *Int J Mol Sci*. 2020;21(11):4012.
2. Rawla P, Barsouk A. Epidemiology of gastric cancer: global trends, risk factors and prevention. *Prz Gastroenterol*. 2019;14(1):26–38.
3. Waddingham W, Nieuwenburg SAV, Carlson S, Rodriguez‐Justo M, Spaander M, Kuipers EJ, et al. Recent advances in the detection and management of early gastric cancer and its precursors. *Frontline Gastroenterol*. 2021;12(4):322–331.
4. Lahner E, Esposito G, Galli G, Annibale B. Atrophic gastritis and pre‐malignant gastric lesions. *Transl Gastrointest Cancer*. 2015;4(4):272–281.
5. Cheung DY. Atrophic gastritis increases the risk of gastric cancer in asymptomatic population in Korea. *Gut Liver*. 2017;11(5):575–576.
6. Agkoc M, Dursun H, Albayrak F, Yilmaz O, Kiziltunc A, Yilmaz A, et al. Usefulness of serum pepsinogen levels as a screening test for atrophic gastritis and gastric cancer. *Eurasian J Med*. 2010;42(1):15–18.
7. Su W, Zhou B, Qin G, Chen Z, Geng X, Chen X, et al. Low PG I/II ratio as a marker of atrophic gastritis: association with nutritional and metabolic status in healthy people. *Medicine (Baltimore)*. 2018;97(20):e10820.
8. Miftahussurur M, Waskito LA, Aftab H, Vilaichone RK, Subsomwong P, Nusi IA, et al. Serum pepsinogens as a gastric cancer and gastritis biomarker in South and Southeast Asian populations. *PLoS One*. 2020;15(4):e0230064.

**Disclosure**: Nothing to disclose.

## LB14 | A COMPUTER‐AIDED DETECTION SYSTEM IN THE EVERYDAY SETTING OF DIAGNOSTIC, SCREENING AND SURVEILLANCE COLONOSCOPY: AN INTERNATIONAL, RANDOMIZED TRIAL

M. H. J. Maas ^1^, T. Rath^2^, C. Spada^3^, E. Soons^1^, N. Forbes^4^, S. V. Kashin^5^, P. Cesaro^6^, A. Eickhoff^7^, G. Vanbiervliet^8^, P. D. Siersema^1,9^

^1^Gastroenterology and Hepatology, Radboud University Medical Center, Nijmegen, Netherlands, ^2^Ludwig Demling Endoscopy Center of Excellence, University Hospital Erlangen, Erlangen, Germany, ^3^Catholic University Rome ‐ Digestive Endoscopy unit, Catholic University Rome; Rome/IT, Digestive Endoscopy Unit, Rome, Italy, ^4^Division of Gastroenterology & Hepatology, University of Calgary, Calgary, Canada, ^5^Department of Endoscopy, Yaroslavl Clinical Oncology Hospital, Yaroslavl, Russia, ^6^Department of Gastroenterology and Digestive Endoscopy, Fondazione Poliambulanza, Brescia, Italy, ^7^Klinikum Hanau, Hanau, Germany, ^8^CHU, Hôpital L'Archet 2 Gastroentérologie ‐ Gastroenterology, CHU, Hôpital L'Archet 2 Gastroentérolo, Gastroenterology, Nice, France, ^9^Gastroenterology and Hepatology, ErasmusMC, Rotterdam, Netherlands

**Contact E‐Mail Address**: Michiel.maas@radboudumc.nl

**Introduction**: Artificial intelligence systems, also known as computer‐aided detection (CADe) systems, have been developed to improve lesion detection rate during colonoscopy. After initial reports of high efficacy, there is an increasing recognition of variability in the effectiveness across different CADe systems. Moreover, recent real‐world studies of commercially available systems reported comparable adenoma detection rates between CADe and conventional colonoscopy (CC). The aim of this study was to evaluate a CADe system, the DISCOVERY^TM^ system (PENTAX Medical, Tokyo, Japan), in a diagnostic, non‐iFOBT screening, and surveillance colonoscopy population.

**Aims & Methods**: A multicenter, randomized, controlled trial (RCT) was conducted at 7 hospitals (both university and non‐university) in Europe and Canada. Patients referred for diagnostic, non‐iFOBT screening, or surveillance colonoscopy were included. Patients were randomized (1:1) to undergo CADe‐assisted colonoscopy or CC by experienced endoscopists (>500 independently performed colonoscopies). Patients with a total Boston Bowel Preparation Scale (BBPS) <6 were excluded from the final analysis. Primary outcome was adenoma detection rate (ADR). Secondary outcomes included mean adenoma per colonoscopy (APC), polyp detection rate (PDR) and mean sessile serrated lesion per colonoscopy (SSLPC).

**Results**: In total, 567 patients were enrolled, of which 497 were included in the final analysis; 250 in the CADe arm and 247 in the CC arm. Surveillance was the indication in 202/497 (40.6%) colonoscopies, followed by diagnostic in 199/497 (40.0%), and non‐iFOBT screening in 96/497 (19.3%), with equal distribution between both study arms. Overall, ADR was similar between CADe versus CC; 38.4% (96/250) versus 37.7% (93/247), *p* = 0.86. APC was also similar between CADe versus CC; 0.66 (166/250) versus 0.66 (163/247), *p* = 0.97. CADe increased PDR, albeit not statistically significantly; 55.2% (138/250) versus 51.4% (127/247), *p* = 0.398. Notably, SSLPC was increased in the CADe group versus CC; 0.30 (76/250) versus 0.19 (46/247), *p* = 0.049.

**Conclusion**: In our study conducted by experienced endoscopists, CADe did not result in a statistically significant increase in adenoma detection (ADR or APC) in patients with variable indications for colonoscopy. Remarkably, the detection of sessile serrated lesions was significantly increased by 58%, suggesting that this CADe system can detect these more subtle lesions which are increasingly recognized for their clinical importance, even when used by experts.

**Disclosure**: Nothing to disclose.

## LB15 | EVALUATION OF ARTIFICIAL INTELLIGENCE‐ASSISTED COLONOSCOPY FOR ADENOMA DETECTION IN LYNCH SYNDROME: A MULTICENTRE RANDOMIZED CONTROLLED TRIAL (TIMELY STUDY)

O. Ortiz ^1^, L. Rivero Sánchez^1^, S. Tejpar^2^, A. Zebenzuy Gimeno^3^, J. López Vicente^4^, F. Riu Pons^5^, R. Hüneburg^6^, A. Huerta Madrigal^7^, R. Jover^8^, M. Carrillo Palau^3^, G. M. Cavestro^9^, L. Ricciardiello^10^, C. Pierantoni^10^, C. Romero Mascarell^11^, A. Repici^12^, C. Hassan^13^, R. Bisschops^14^, M. Daca Alvarez^15^, J. Nattermann^16^, J. M. Herrero Rivas^17^, D. Remedios^18^, M. V. Alvarez Sanchez^19^, A. Ledo^20^, J. Gordillo^21^, I. Puig^22^, M. Herraiz Bayod^23^, C. Alvarez Urturi^24^, M. T. Betes Ibañez^25^, M. Puzzono^26^, L. Cid^27^, F. Balaguer^1^, M. Pellisé Urquiza^28^

^1^Gastroenterology, Hospital Clínic of Barcelona, Barcelona, Spain, ^2^Department of Internal Medicine, AZ. Gasthuisberg, Leuven, Belgium, ^3^Hospital de Canarias, Santa Cruz de Tenerife, Spain, ^4^Hospital Universitario de Móstoles, Madrid, Spain, ^5^Gastroenterology Department, Hospital del Mar, Barcelona, Spain, ^6^Department of Internal Medicine I/National Center for Hereditary Tumor Syndromes, University Hospital Bonn, Bonn, Germany, ^7^Gastroenterology Service, H. Galdakao‐Usansolo, Galdakao, Spain, ^8^Hospital Universitario de Alicante, Alicante, Spain, ^9^Division of Gastroenterology and Gastrointestinal Endoscopy, Univerdità San Raffele, Milan, Italy, ^10^Medical and Surgical Sciences, University of Bologna, Bologna, Italy, ^11^Hospital Clínic of Barcelona, Endo Tecnica S.L., Madrid, Spain, ^12^Department of Gastroenterology, Department of Gastroenterology, Ist. Clinico Humanitas Rozzano, Milano, Italy, ^13^Gastroenterology, Humanitas University, Rome, Italy, ^14^Gastroenterology, Katholieke Universiteit Leuven, Leuven, Belgium, ^15^Hospital Clínic of Barcelona, Barcelona, Spain, ^16^National Center for Hereditary Tumor Syndromes (NZET), Bonn, Germany, ^17^Gastroenterology, Hospital de Ourense, Ourense, Spain, ^18^Hospital de Ourense, Ourense, Spain, ^19^Gastroenterology, Complejo Hospitalario Universitario de Pontevedra, Pontevedra, Spain, ^20^Laboratorios Salvat, S.a., Digestive Diseases, Esplugues De Llobregat, Spain, ^21^Gastroenterology Unit, Hospital de la Santa Creu i Sant Pau, Barcelona, Spain, ^22^Digestive Diseases Department, Althaia, Xarxa Assistencial Universitària de Manresa, Manresa, Spain, ^23^University of Navarra, Navarra, Spain, ^24^Gastroenterology, H. del Mar ‐ Parc de Salut Mar, Barcelona, Spain, ^25^Clinica Universidad de Navarra, Navarra, Spain, ^26^Gastroenterology and Gastrointestinal Endoscopy Unit, Division of Experimental Oncology, Vita‐Salute, Milan, Italy, ^27^Complejo Hospitalario de Vigo, Vigo, Spain, ^28^Hospital Clinic, Gastroenterology, Barcelona, Spain

**Contact E‐Mail Address**: oswaldoaoz@gmail.com

**Introduction**: Colorectal cancer (CRC) risk reduction through colonoscopy is a well‐documented strategy within Lynch syndrome (LS), a genetic disorder characterized by 50%–70% lifetime CRC susceptibility. In LS, CRC arises from small, proximal, and flat adenomas, following an accelerated adenoma‐carcinoma sequence. Although artificial intelligence‐assisted colonoscopy (CADe) has shown to increase adenoma detection among individuals at average risk, its efficacy within the LS context remains unexplored.

**Aims & Methods**: A prospective, parallel, randomized, multicenter study was conducted to compare CADe‐assisted colonoscopy (intervention) with standard white‐light endoscopy (WLE, control) in individuals harboring pathogenic/likely pathogenic MLH1, MSH2, MSH6, or EpCam variants associated with LS. The mean adenomas detected per colonoscopy (APC), guided the sample size calculation and primary outcome.

**Results**: Out of the initially randomized 430 individuals, 414 patients were included for final analysis: 204 in the intervention group and 210 in the control group. Baseline characteristics were well‐distributed between the two groups. Comparable mean withdrawal times were observed for CADe (12.5 min) and WLE (13.1 min; *p* = 0.3). Adenoma detection rate was 35.3% in the whole cohort (CADe: 33.3% vs. WLE: 37.1%; *p* 0.42). The overall APC and the APC categorized by adenoma size, location, and morphology did not exhibit statistically significant differences between groups. CADe demonstrated a significant increase in the serrated lesions detection rate (38.7% vs. 26.7%; Relative risk 1.31 [1.08–1.59]; *p* 0.01) and in the number of 5–9 mm serrated lesions per colonoscopy (Table 1). Based on the histology results, the CADe group exhibited a higher count of resected lesions with normal mucosa compared to the WLE group (0.21 [0.67] vs. 0.08 [0.30]; *p* < 0.04).

**TABLE 1:** Performance between WLE and CADe.

	CADe = 204	WLE = 210	Relative risk (95% CI)	*p* value
Adenomas per colonoscopy (APC): Mean, (SD)	0.64 (1.59)	0.64 (1.17)	1 (0.73–1.27)	0.42
<5 mm	0.41 (0.96)	0.37 (0.79)	1.11 (0.91–1.24)	0.87
5–9 mm	0.21 (0.71)	0.22 (0.59)	0.95 (0.84–1.09)	0.46
Advanced	0.03 (0.18)	0.05 (0.24)	0.6 (0.37–0.74)	0.49
Flat (IIa‐IIb‐IIc)	0.43 (1.06)	0.35 (0.70)	0.84 (0.58–1.73)	0.34
Serrated lesions per colonoscopy: Mean, (SD)	0.58 (0.95)	0.46 (0.94)	1.26 (0.96–1.32)	**0.02**
<5 mm	0.42 (0.83)	0.38 (0.79)	1.11 (0.91–1.22)	0.36
5–9 mm	0.15 (0.45)	0.06 (0.24)	2.5 (2.35–2.52)	**0.04**
Advanced	0.01 (0.09)	0.03 (0.18)	0.33 (0.31–0.37)	0.27

**Conclusion**: CADe did not improve the detection of adenomas in Lynch syndrome in referral centers with a high adenoma detection rate.

**Disclosure**: Nothing to disclose.

## AI and machine learning in gastroenterology: Late‐breaking abstracts Tuesday, 17 October 2023 14:30–15:30/A1

### LB16 GASTROGPT: FIRST SPECIALTY‐SPECIFIC AI LANGUAGE MODEL OUTPERFORMS GENERAL MODELS ACROSS KEY CLINICAL TASKS

C. Simsek ^1^, E. de‐Madaria^2^, A. Ebigbo^3^, P. Vanek^4^, O. Elshaarawy^5^, A. M. Voiosu^6^, G. Antonelli^7^, R. Turro^8^, J. Gisbert^9^, O. P. Nyssen^9^, C. Hassan^10^, H. Messmann^11^, R. Jalan^12^, H. Demir^13^, B. Tinaz^14^, H. M. Erol^15^

^1^Division of Gastroenterology, Hepatology and Endoscopy, Hacettepe University, Ankara, Turkey, ^2^Gastroenterology, Hospital General Universitario Dr. Balmis, Alicante, Spain, ^3^Klinikum Augsburg, III. Medizinische Klinik, Augsburg, Germany, ^4^2nd Department of Medicine ‐ Gastroenterology and Geriatrics, University Hospital Olomouc, Olomouc, Czech Republic, ^5^Gastroenterology and Hepatology, Royal Liverpool University Hospital, Liverpool, UK, ^6^Department of Gastroenterology, Colentina Clinical Hospital, Bucharest, Romania, ^7^Digestive and Liver Diseases Unit, Sapienza Università di Roma at Sant'Andrea University Hospital, Roma, Italy, ^8^Endoscopy, Centro Medico Teknon Unidad Endoscopia Digestiva, Barcelona, Spain, ^9^Hospital Universitario de La Princesa, Instituto de Investigación Sanitaria Princesa and CIBEREHD, Digestive Services, Madrid, Spain, ^10^Gastroenterology, Humanitas University, Rome, Italy, ^11^Uniklinik Augsburg, III. Medizinische Klinik, Augsburg, Germany, ^12^The Royal Free Hospital London ‐ UCL Medical School, The Royal Free Hospital London, UCL Medical School, London, UK, ^13^AudioEye, Tucson, USA, ^14^University of Southern California, Los Angeles, USA, ^15^Massachusetts Institute of Technology, Cambridge, USA

**Contact E‐Mail Address**: cemgsimsek@gmail.com

**Introduction**: Currently available artificial intelligence (AI) large language models (LLM) fail to translate effectively to clinical medicine, largely being relegated to question answering roles, documentation, and medical literature summarization. Recognizing this potential, we developed a proof of concept of a specialty‐specific and multi‐task clinical LLM: GastroGPT. Our study evaluated GastroGPT against leading general‐purpose LLMs across key clinical tasks and diverse gastroenterology case scenarios.

**Aims & Methods**: In this structured analysis, GastroGPT was compared to three state‐of‐the‐art general purpose LLMs (LLM‐A: GPT4, LLM‐B: Bard, LLM‐C: Claude) with highest known utility metrics (training and model parameters, published performance benchmarks, user adoption). All models were assessed on seven key clinical tasks and overall performance. Ten simulated cases covered general gastroenterology and sub‐specialties across varying complexity, frequency, and demographics. Standardized prompts ensured structured comparisons. Expert panels, representing varied backgrounds, blindly rated model outputs overall and per task using a 10‐point Likert scale, judging utility, style, practicality, and knowledge depth. 480 expert scorings were obtained. Comprehensive statistical analyses were performed.

**Results**: GastroGPT significantly outperformed other LLMs overall (8.05 ± 1.9 vs. 4.95 ± 2.95, 5.63 ± 3.35, 6.92 ± 2.69; *p* < 0.001). GastroGPT also scored higher in models overall comparison in each case (*p* < 0.05). GastroGPT demonstrated superior performance in six of seven tasks: (1) Initial assessments (*p* < 0.001 vs. LLM‐A and LLM‐B, *p* = 0.39 vs. LLM‐C), (2) Additional history gathering (*p* < 0.001 for all), (3) Diagnostic test recommendations (*p* < 0.001 vs. LLM‐A, *p* = 0.83 vs. LLM‐B, *p* = 0.35 vs. LLM‐C), (4) Management plans and treatment strategies (*p* < 0.001 vs. LLM‐A and C, *p* = 0.57 vs. LLM‐B), (6) Multidisciplinary care organization and specialist referrals (*p* < 0.001 vs. all) and (7) Patient education and counseling (*p* < 0.001 for LLM‐A and B, *p* = 0.07 vs. LLM‐C). In task (5) follow‐up planning LLM‐B scored comparably (7.45 ± 2.04 vs. 7.82 ± 1.88, *p* = 0.35) (Table 1). GastroGPT showed more consistent performance across cases (Levene's test *p* < 0.001 for all tasks). Subgroup analysis illustrated that GastroGPT showed higher performances across different complexity levels and frequencies. In multivariable analysis accounting for complexity, frequency, and clinical task, LLM choice emerged as a significant predictor of performance (*p* < 0.001). Reviewer panel scores showed acceptable consistency (Cronbach's alpha: 0.76 CI: 0.71–0.79).

**TABLE 1:** Evaluation scores for models' performances in key clinical tasks.

	GastroGPT	LLM‐A	LLM‐B	LLM‐C	*p*
Task 1 ‐ initial assessments	**8.02 ± 1.73** ^**A,B**^	5.83 ± 2.67	6.72 ± 2.23	7.75 ± 1.78	<0.001
Task 2 ‐ additional history gathering	**8.38 ± 1.96** ^**A,B,C**^	2.90 ± 3.04	2.85 ± 3.24	2.72 ± 2.92	<0.001
Task 3 ‐ diagnostic tests	**7.75 ± 1.83** ^**A**^	4.32 ± 3.32	7.33 ± 2.82	7.52 ± 1.70	<0.001
Task 4 ‐ management plans	**7.85 ± 2.22** ^**A,C**^	6.27 ± 2.21	7.65 ± 2.23	6.20 ± 2.98	<0.001
Task 5 ‐ follow‐up planning	7.45 ± 2.04^A,B^	5.55 ± 2.51	4.20 ± 3.47	**7.82 ± 1.88** ^**A,B**^	<0.001
Task 6 ‐ multidisciplinary care, referral	**8.25 ± 1.74** ^**A,B,C**^	4.25 ± 3.36	5.12 ± 3.48	7.72 ± 1.71	<0.001
Task 7 ‐ patient education and counseling	**8.40 ± 1.83** ^**A,B**^	4.97 ± 2.73	4.93 ± 3.29	7.83 ± 1.90	<0.001
Task 8 ‐ overall evaluation	**8.30 ± 1.28** ^**A,B,C**^	5.58 ± 2.02	6.23 ± 2.16	7.78 ± 1.42	<0.001

*A: *p* < 0.05 for LLM‐A, B: *p* < 0.05 for LLM‐B, C: *p* < 0.05 for LLM‐C. **LLM‐A: GPT4, LLM‐B: Bard, LLM‐C: Claude. ***Highest mean scores in each task are highlighted. LLM: Large language model.

**Conclusion**: Our study pioneers the development and comparison of a specialty‐specific, clinically oriented AI model to state‐of‐the art general‐purpose LLMs. GastroGPT demonstrated superior utility overall and in key clinical tasks of gastroenterology practice. These findings underscore the potential for specialty‐focused, clinically‐ and task‐oriented AI models in medicine.

**Disclosure**: Olga P Nysse: IDEM.

Rajiv Jalan: Inventor of OPA, which has been patented by UCL and licensed to Mallinckrodt Pharma. He is also the founder of Yaqrit Discovery, a spin out company from University College London, Hepyx Limited and Cyberliver (the company that holds the intellectual property for AlcoChange). He had research collaborations with Yaqrit Discovery

## LB17 | THE EFFECTIVENESS OF AN APP‐BASED DIGITAL THERAPEUTIC FOR IRRITABLE BOWEL SYNDROME: RESULTS OF A RANDOMIZED CONTROLLED TRIAL

L. M. Weißer ^1^, D. Zhao^1^, A. Sommer^1^, A. Mocek^2^, M. Storr^3^

^1^HiDoc Technologies GmbH, Hamburg, Germany, ^2^IGES Institut GmbH, Berlin, Germany, ^3^Center of Endoscopy, Starnberg, Germany

**Contact E‐Mail Address**: linda.weisser@gmail.com

**Introduction**: Treatment of irritable bowel syndrome (IBS) comprises a multidisciplinary approach that is tailored to an individual patient, including educative, psychotherapeutic, and dietary interventions. Due to several limiting factors, including limited resources, currently not all IBS patients receive adequate treatment.

Digital health applications (DiGAs) are predominantly app‐based digital therapeutics controlled by the German Federal Institute for Drugs and Medical Devices (BfArM). By providing guided self‐help, DiGAs are considered promising treatment options for chronic conditions such as IBS, thereby closing gaps in healthcare.

**Aims & Methods**: The aim of this study was to prove the superiority of a BfArM registered DiGA intended to treat IBS compared to waiting list.

378 adults with diagnosed IBS were randomized to either using the DiGA for 12 weeks (intervention group, *n* = 188), or to using a modified sham app that only contained the measuring instruments, i.e., questionnaires used for this study (control group, *n* = 190). Both groups had access to uncontrolled add on care ad libitum.

The DiGA provided an in‐app symptom journal and a personalized treatment plan containing recommendations from the fields of nutrition therapy (low‐FODMAP diet) and psychotherapy (psychoeducation, cognitive‐behavioral therapy, or gut‐directed hypnosis).

The primary outcomes were symptom severity reduction, measured using the Irritable Bowel Syndrome ‐ Severity Scoring System (IBS‐SSS), quality of life improvement, measured using the Irritable Bowel Syndrome ‐ Quality of Life (IBS‐QOL) questionnaire, work productivity impairment reduction & activity impairment reduction, measured using the Work Productivity and Activity Impairment: Irritable Bowel Syndrome (WPAI:IBS) questionnaire, and health literacy improvements, measured using the European Health Literacy Survey (HLS‐EU‐Q16).

Outcomes were assessed at baseline and at week 12 (postintervention). The primary analysis was based on the intention‐to‐treat principle and used linear mixed models. An improvement of at least 50 points on the IBS‐SSS scale was considered clinically relevant.

**Results**: Patients randomized to use the DiGA experienced statistically significant greater improvement compared to patients randomized to the control group for symptom severity (*p* < 0.001; 95% CI −123.9, −79.9), quality of life (*p* < 001; 95% CI 16.2, 22.1), work productivity impairment (*p* < 0.001; 95% CI −0.2, −0.1), activity impairment (*p* < 0.001; 95% CI −0.2, −0.1), and health literacy (*p* < 0.001; 95% CI 0.7, 1.8) after 12 weeks (postintervention).

66.5% of the intervention group patients (125/188) had a clinically relevant improvement of symptoms, evaluated by IBS‐SSS, compared to 33.2% of the control group (63/190, odds ratio 4.871; *p* < 0.001; 95% CI 3.122, 7.601; number needed to treat = 3).

**Conclusion**: The investigated DiGA was found to be largely effective in patients with IBS. It provides a treatment option that produces a significant, clinically relevant reduction of symptoms as well as significant improvements of quality of life, work productivity, and health literacy. Further studies should determine what long‐term effects can be achieved, and how the use of the DiGA impacts health economic variables such as treatment costs for IBS.

**Disclosure**: Linda M. Weißer and André Sommer are employees of HiDoc Technologies GmbH.

Dongxing Zhao is a former employee of HiDoc Technologies GmbH.

Anja Mocek is an employee of IGES Institut GmbH.

Martin Storr has served as consultant for HiDoc Technologies GmbH.

The study was designed by IGES Institut GmbH and carried out by CSG Clinische Studien GmbH.

Funding was provided by HiDoc Technologies GmbH.

## LB18 | COLO‐DETECT: A RANDOMISED CONTROLLED TRIAL OF POLYP DETECTION COMPARING COLONOSCOPY ASSISTED BY THE GI GENIUS™ ARTIFICIAL INTELLIGENCE ENDOSCOPY MODULE WITH STANDARD COLONOSCOPY

A. Seager ^1,2^, L. Sharp^2^, J. S. Hampton^1,2^, L. J. Neilson^3^, T. J. Lee^2,4^, A. Brand^5^, R. Evans^5^, L. Vale^6^, J. Whelpton^7^, C. J. Rees^2,3^

^1^South Tyneside and Sunderland NHS Foundation Trust, Research and Innovation, South Shields, UK, ^2^Newcastle University, Population Health Sciences Institute, Newcastle Upon Tyne, UK, ^3^Gastroenterology, South Tyneside and Sunderland NHS Foundation Trust, South Shields, UK, ^4^Gastroenterology, Northumbria Healthcare NHS Foundation Trust, North Shields, UK, ^5^North Wales Organisation for Randomised Trials in Health, Bangor, UK, ^6^Newcastle University, Health Economics Group, Newcastle Upon Tyne, UK, ^7^Patient & Public Involvement Representative, Newcastle Upon Tyne, UK

**Contact E‐Mail Address**: alexander.seager@nhs.net

**Introduction**: Reducing polyp miss rate (PMR) during colonoscopy is imperative in reducing post‐colonoscopy colorectal cancer incidence. Artificial Intelligence‐based Computer‐Aided Detection (CADe) systems are intended to support colonoscopists to reduce PMR. The GI Genius™ intelligent endoscopy module (Medtronic ltd.) is a CADe system, compatible with endoscopy systems from most manufacturers.

**Aims & Methods**: COLO‐DETECT assessed clinical effectiveness of the GI Genius™ intelligent endoscopy module in routine colonoscopy practice by comparing GI Genius™‐assisted colonoscopy (GGC) with standard colonoscopy (SC).

COLO‐DETECT was a multi‐site parallel‐arm randomised controlled trial, undertaken in 10 English NHS Trusts, amongst adults attending for colonoscopy for the indications of colorectal cancer screening (screening population), or gastrointestinal symptoms/surveillance (symptomatic population). Randomisation was one‐to‐one, stratified by age category (<60 years, 60–73 years, ≥74 years), sex, colonoscopy indication, and NHS Trust. The trial statistician and co‐chief investigators were blinded to allocation.

Primary outcome was mean adenomas per procedure (MAP); key secondary outcome was adenoma detection rate (ADR). Primary analysis was intention‐to‐treat.

A patient and public involvement representative contributed to all stages of the trial. COLO‐DETECT was prospectively registered (ISRCTN: 10451355, ClinicalTrials.gov: NCT04723758, NIHR Portfolio ID: 48022). The protocol was prospectively published.

COLO‐DETECT was funded by Medtronic ltd. who had no role in trial design, delivery, or reporting.

**Results**: 2032 participants were recruited between 29/03/21 and 06/04/23: 28% were <60 years, 61.5% were 60–73 years, and 10.5% were ≥74 years; 55.7% were male; 60.6% were undergoing bowel cancer screening. Randomisation allocation was equal (1015 to GGC, 1017 to SC).

MAP for GGC was 1.56 (SD 2.82) versus 1.12 (SD 1.91) for SC (IRR 1.30; 95% CI 1.15–1.47; *p* < 0.001). ADR was 56.6% (GGC) versus 48.4% (SC), (OR 1.47; 95% CI 1.21–1.78; *p* < 0.001).

GGC demonstrated smaller mean polyp size (5.18 mm vs. 5.78 mm; 95% CI −1.08 to −0.11; *p* = 0.016), more 0‐IIa polyps (34% GGC vs. 27% SC; *χ* ^2^(5) = 19.34, *p* = 0.002), and higher sessile serrated polyp (SSP) detection rate (11.6% vs. 8.3%; OR 1.49; 95% CI 1.09–2.03; *p* = 0.012).

Within the screening population MAP was 2.08 (SD 3.36) in the GGC group versus 1.61 (SD 2.16) in the SC group (IRR 1.29; 95% CI 1.12–1.48; *p* < 0.001), and ADR was 68% in the GGC group versus 61% in the SC group (OR 1.37; 95% CI 1.07–1.74; *p* = 0.011).

Within the symptomatic population MAP was 0.76 (SD 1.33) in the GGC group versus 0.57 (SD 1.18) in the SC group (IRR 1.33; 95% CI 1.03–1.71; *p* = 0.027) and ADR was 39% in the GGC group versus 28.5% in the SC group (OR 1.65; 95% CI 1.2–2.26; *p* = 0.002).

Advanced adenoma detection rate and cancer detection rate did not differ between GGC and SC groups overall, or within the screening or symptomatic populations separately.

Colonoscopic adverse events were equally distributed between the trial arms with none related to the trial intervention.

**Conclusion**: GI Genius™ increases MAP and ADR in routine colonoscopy practice, in all patients as well as symptomatic and screening populations separately. Increased detection is of smaller, flat polyps, which are more likely to be missed during colonoscopy. Detection of SSPs was increased, which are over‐represented among lesions causing post‐colonoscopy colorectal cancer. These increases occurred despite high baseline MAP and ADR in the control arm. COLO‐DETECT supports the adoption of GI Genius™ into colonoscopy practice to increase detection of premalignant polyps.

**Disclosure**: Nothing to disclose.

## LB19 | SCREENING MODIFIED BY ARTIFICIAL INTELLIGENCE IN ENDOSCOPY (SMARTIE): A MULTICENTER RANDOMIZED CLINICAL TRIAL

T. J. Lux^1^, Z. Saßmannshausen^1^, M. Banck^1,2^, A. Krenzer^1,2^, D. Fitting^1^, B. Sudarevic^1,3^, I. Kafetzis^1^, J. Troya^1^, W. Böck^4^, F. Passek^5^, T. Heubach^6^, B. Simonis^7^, F. Heil^8^, L. Ludwig^9^, F. Puppe^2^, W. Zoller^3^, A. Meining^1^, A. Hann ^1^

^1^Internal Medicine II, University Hospital Würzburg, Würzburg, Germany, ^2^Institute for Computer Science, Artificial Intelligence and Knowledge Systems, Julius‐Maximilians University of Würzburg, Würzburg, Germany, ^3^Department of Internal Medicine and Gastroenterology, Katharinenhospital, Stuttgart, Germany, ^4^Gastroenterological Practice Dres. Boeck/Haegele, Ulm, Germany, ^5^Gastroenterological Practice Bad Saulgau, Bad Saulgau, Germany, ^6^Gastroenterological Practice Heubach‐Begetopoulos, Waiblingen, Germany, ^7^Gastroenterological Practice Darmstadt, Darmstadt, Germany, ^8^Gastroenterological Practice Andernach, Andernach, Germany, ^9^Gastrointestinal practice Dornstadt, Dornstadt, Germany

**Contact E‐Mail Address**: hann_a@ukw.de

**Introduction**: Literature suggests that computer‐aided polyp detection (CADe) systems increase adenoma detection rate (ADR) compared to traditional colonoscopy (TC). Multiple systems with comparable efficacy on benchmark datasets have been introduced, including the for research purposes freely available system EndoMind. Although intended for colorectal cancer screening, many studies have been performed in a hospital setting recruiting mostly non‐screening patients. Additionally, more recent studies present conflicting data suggesting a less pronounced effect of CADe on ADR.

**Aims & Methods**: In this randomized clinical trial we aimed to analyze the impact of the CADe system EndoMind on ADR for screening colonoscopy in a multicenter fashion. Adult patients with an indication for colorectal cancer screening, post‐polypectomy surveillance or with a positive fecal immunochemical test were eligible for participation in five outpatient treating centers in Germany. Exclusion criteria were inflammatory bowel disease, familial polyposis syndrome, previous radiation, or colorectal surgery. Participants were randomized prior to the intervention to the CADe or the TC group. In the first group, the EndoMind CADe system framed detected polyps with a blue box on the main examination monitor during the whole procedure. Colonoscopies were performed by examiners with over 10 years of experience. Primary outcome was ADR. Secondary outcomes included polyp detection rate and withdrawal time. ClinicalTrials.gov trial register number: NCT05006092.

**Results**: From November 2021 to November 2022, 928 subjects were randomized to the CADe (*n* = 457) and TC (*n* = 471) group. Baseline characteristics of patients and bowel preparation quality between both groups were similar. The rate of screening examinations was over 80% in both groups. The mean ADR was 36.8% and 33.8% for the CADe and TC group respectively (*p* = 0.34). Polyp detection rate was 50.8% in the CADe group and 50.5% in the TC group (*p* = 0.94). Mean withdrawal time in total and without time spent on resection was 7.5 min, 6.9 versus 7.4 min, 6.9 min for the CADe and TC group respectively.

**Conclusion**: In our multicenter randomized clinical trial the usage of CADe did not improve ADR or polyp detection rate of expert endoscopists in an outpatient setting involving mainly colorectal cancer screening candidates. The evaluation of other systems in this setting is needed in order to estimate the value of CADe.

**Disclosure**: Nothing to disclose.

## LB20 | MACHINE LEARNING‐BASED PRECISION MEDICINE APPROACH IDENTIFIES SPECIFIC RESPONDER SUB‐POPULATIONS BY DYNAMIC CLUSTERING OF PRO DATA: POST HOC ANALYSIS OF VARSITY DATASET

S. Schreiber ^1^, D. Ostanin^2^, J. Cheng^3^, C. Agboton^4^, V. Yajnik^5^, P. Thakker^2^

^1^University Hospital Schleswig‐Holstein, Kiel, Germany, ^2^Takeda, Translational Medicine and Biomarker Research, Gastrointestinal and Inflammation Drug Discovery Unit, Cambridge, USA, ^3^Takeda, Statistical and Quantitative Sciences, Cambridge, USA, ^4^Takeda, Global Medical Affairs, Cambridge, USA, ^5^Takeda, US Medical Affairs, Cambridge, USA

**Contact E‐Mail Address**: s.schreiber@ikmb.uni‐kiel.de

**Introduction**: In UC, biologics and other targeted therapies generally show overall remission rates of <50% after 1 year using regulatory endpoints, as defined by patient reported outcomes (PRO): rectal bleeding and stool frequency in conjunction with endoscopic improvement. For IBD patients, achieving full control of symptoms quickly after therapy start leads to better long‐term outcomes. Traditional landmark endpoints to define success of treatment have shown mixed success in the real world. Machine learning approaches using dynamic individual patient‐level data could be better suited to derive patterns and to identify responder sub‐populations early in the treatment paradigm. We used VARSITY (NCT02497469) dataset (1) to derive trajectories of response to vedolizumab (VDZ) and adalimumab (ADA) in conjunction with a disease control (DC) endpoint at week 52. Our aim was to use an unbiased clustering to deconvolute the clinical phenotype of UC under two therapies to identify common patterns of response delivered by each therapeutic intervention.

**Aims & Methods**: DC at week 52 was previously defined as a combination endpoint of clinical, endoscopic and histological response with CRP/FcP normalization (2, 3). The full set of daily patient‐reported daily outcome recordings over 52 weeks was subjected to an unsupervised machine learning algorithm (Group‐Based Multivariate Trajectory Modeling). The BIC criterion was used to determine the optimal number of clusters. After visual examination of disease trajectories, we manually merged similar trajectories.

**Results**: We identified four distinct patient clusters in the dynamic PRO data collected over 52 weeks: Super‐Response (SR), Response (R), Partial Response (PR) and Incomplete/Non‐Response (INR). In SR and R clusters, up to 30% of patients reached DC, regardless of treatment, whereas patients in PR and INR groups had almost no chance to reach DC. SR and R groups in the VDZ arm had twice as many patients who reached DC at week 52 compared with ADA (*n* = 39 vs. *n* = 17, respectively). SR was seen 5 times more frequently with VDZ (170 patients [pts]) than with ADA treatment (27 pts). Our findings confirm previous supervised analyses, in which early remission at week 6 predicts remission and DC at week 52 under VDZ but not under ADA.

**Table:** 

Treatment	Vedolizumab				Adalimumab			
Longitudinal clusters	Super‐response	Response	Partial response	Incomplete/Non‐response	Super‐response	Response	Partial response	Incomplete/Non‐response
*N*	170	42	101	69	27	80	257	22
SEX = F	73 (43%)	15 (36%)	37 (37%)	26 (38%)	15 (56%)	36 (45%)	112 (44%)	7 (32%)
SEX = M	97 (57%)	27 (64%)	64 (63%)	43 (62%)	12 (44%)	44 (55%)	145 (56%)	15 (68%)
TNF = failure	33 (19%)	8 (19%)	21 (21%)	17 (25%)	3 (11%)	11 (14%)	59 (23%)	8 (36%)
TNF = Naïve	137 (81%)	34 (81%)	80 (79%)	52 (75%)	24 (89%)	69 (86%)	198 (77%)	14 (64%)
Corticosteroids = no	120 (71%)	30 (71%)	61 (60%)	34 (49%)	16 (59%)	53 (66%)	163 (63%)	14 (64%)
Corticosteroids = yes	50 (29%)	12 (29%)	40 (40%)	35 (51%)	11 (41%)	27 (34%)	94 (37%)	8 (36%)
Endoscopic = moderate	66 (39%)	14 (33%)	39 (39%)	26 (38%)	16 (59%)	32 (40%)	86 (33%)	6 (27%)
Endoscopic = severe	103 (61%)	28 (67%)	62 (61%)	43 (62%)	11 (41%)	48 (60%)	171 (67%)	16 (73%)
Duration <1 year	20 (12%)	3 (7%)	17 (17%)	6 (9%)	4 (15%)	10 (13%)	21 (8%)	4 (18%)
Duration 1–<3 years	35 (21%)	15 (36%)	20 (20%)	21 (30%)	10 (37%)	16 (20%)	62 (24%)	3 (14%)
Duration 3–<7 years	50 (29%)	10 (24%)	24 (24%)	22 (32%)	6 (22%)	21 (26%)	92 (36%)	7 (32%)
Duration ≥7 years	65 (38%)	14 (33%)	40 (40%)	20 (29%)	7 (26%)	32 (40%)	82 (32%)	8 (36%)
Rectal bleeding = 0, 1	52 (31%)	10 (24%)	50 (50%)	35 (51%)	6 (22%)	23 (29%)	5 (2%)	11 (50%)
Rectal bleeding = 2, 3	118 (69%)	31 (74%)	50 (50%)	33 (48%)	21 (78%)	57 (71%)	100 (39%)	9 (41%)
Stool frequency = 0, 1	25 (15%)	1 (2%)	23 (23%)	12 (17%)	5 (19%)	4 (5%)	60 (23%)	2 (9%)
Stool frequency = 2	73 (43%)	15 (36%)	49 (49%)	28 (41%)	14 (52%)	37 (46%)	103 (40%)	3 (14%)
Stool frequency = 3	72 (42%)	25 (60%)	28 (28%)	28 (41%)	8 (30%)	39 (49%)	89 (35%)	15 (68%)

**Conclusion**: We demonstrate that the dynamics of the disease course under therapy can be used to identify meaningful subpopulations in UC. The observed heterogeneity cannot be explained by baseline demographic/disease characteristics and may represent different pathophysiologies of UC. Super‐responders have the highest chances to achieve DC. The large level of differentiation between ADA and VDZ highlights the power of the analysis to discriminate drug efficacies in a comparative setting. If super‐response is not achieved under ADA, patients might be switched to other therapies as early as after 4 weeks. Further work is necessary to prospectively investigate response trajectories in different MoAs. Eventually, disease trajectories may become an important monitoring tool for personalized medicine and clinical decision‐making.

**References**

1. Sands BE, Sandborn WJ, Panaccione R, O’Brien CD, Zhang H, Johanns J, et al. Ustekinumab as induction and maintenance therapy for ulcerative colitis. *N Engl J Med*. 2019;381:1215–1226.
2. Danese S, et al. *J Crohns Colitis*. 2021;15:S305.
3. Schreiber S, et al. *J Crohns Colitis*. 2023;17:863–875.

**Disclosure**: Funding: European Commission (3TR), Excellence Cluster PMI and Takeda.

S. Schreiber reports consultancy and personal fees from AbbVie, Arena, BMS, Biogen, Celltrion, Celgene, Dr Falk, Ferring, Fresenius, Gilead, HIKMA, IMAB, Janssen, MSD, Mylan, Pfizer, Protagonist, Provention Bio, Sandoz, Takeda and Theravance.

D. Ostanin, J. Cheng, C. Agboton, V. Yajnik and P. Thakker are employees of Takeda and hold stock/stock options in Takeda.

